# Coptisine regulates PI3K/AKT pathway to block bladder cancer progression: a study based on network pharmacology, in vitro and in vivo assays

**DOI:** 10.1186/s41065-025-00600-7

**Published:** 2025-11-24

**Authors:** Yu Xiaohui, Li Jie, Zhou Jiangqiao

**Affiliations:** 1 Department of Vascular Surgery, Renmin Hospital of Wuhan University, Wuhan, China; 2 Department of Urology, Renmin Hospital of Wuhan University, Wuhan, China; 3 Department of Organ Transplantation, Renmin Hospital of Wuhan Univaersity, Wuhan University, Wuhan, Hubei China

**Keywords:** Coptisine, Bladder cancer, Network pharmacology, Molecular docking, PI3K/AKT pathway

## Abstract

**Background:**

Coptisine (COP) is a natural compound extracted from *Rhizoma Coptidis*, and it represses the malignant biological behaviors of bladder cancer cells. However, the underlying molecular mechanism has not been fully elucidated. The aim of this study was to clarify the downstream mechanism by which COP treats bladder cancer.

**Materials and methods:**

SwissTargetPrediction, STITCH, SymMap, ETCM, TCMSP, CTD databases were used to collect the related targets of COP. GeneCards, DisGeNET, TTD and OMIM databases were used to obtain the related targets of bladder cancer. A Venn diagram was used to identify the potential targets of COP in bladder cancer treatment. The protein-protein interaction network was constructed using STRING database, and Cytoscape 3.9.0 software was used to screen the hub targets. The binding relationship between COP and the hub targets was verified by molecular docking and molecular dynamics simulation. After the bladder cell lines T24 and BIU-87 were treated with different doses of COP, the regulatory effects of COP on PI3K/AKT pathway were investigated with western blotting. Additionally, the tumor-suppressive properties of COP on bladder cancer cells were validated with tumorigenesis model and metastasis model in nude mice.

**Results:**

RAC-alpha serine/threonine-protein kinase 1 (AKT1), glycogen synthase kinase 3 beta (GSK3B), caspase-3 (CASP3), tumor necrosis factor (TNF) and cyclin D1 (CCND1) were identified as the main hub targets of COP in bladder cancer treatment. PI3K/AKT pathway was predicted to be a crucial pathway regulated by COP. The binding affinities between COP and AKT1, GSK3B, CASP3, TNF and CCND1 were high. COP treatment markedly repressed the phosphorylation level of ERK1/2, AKT1, PI3K p85 and mTOR in T24 and BIU-87 cells, and repressed the tumorigenesis and lung/liver metastasis of T24 cells in vivo.

**Conclusion:**

COP may be a natural inhibitor for AKT1, GSK3B, CASP3, TNF and CCND1. COP represses PI3K/AKT pathway to suppress the progression of bladder cancer.

**Supplementary Information:**

The online version contains supplementary material available at 10.1186/s41065-025-00600-7.

## Introduction

Bladder cancer is one of the most common malignant tumors of urinary system in the world, and also an important cause of cancer-related death. Globally in 2020, there were 573,278 new cases and 212,536 deaths from bladder cancer [1, 2]. The most important risk factor for bladder cancer is smoking [3]. Non-muscle-invasive bladder cancer (NMIBC) is the main pathological type of bladder cancer, accounting for about 75% of all cases, while muscle-invasive bladder cancer (MIBC) accounts for 25–30% of cases [4]. Currently, surgery is the main treatment for NMIBC. However, nearly 50%-80% of patients experience recurrence after resection [5]. Chemotherapy is a common treatment for advanced bladder cancer [6]. However, the development of cisplatin resistance has also become a major obstacle [7]. Therefore, there is an urgent need to develop new and effective treatments for bladder cancer.

Natural drugs have shown great potential in treating human diseases, such as endocrine and metabolic diseases, cardiovascular diseases, inflammatory diseases and tumors [8]. Coptisine (COP) is a natural isoquinoline alkaloid found in *Rhizoma Coptidis* (Huanglian). *Rhizoma Coptidis* is a widely used traditional Chinese herbal medicine [9]. COP has antiviral, antioxidant and anti-inflammatory pharmacological effects, as well as potential cardiovascular and renal protective activities [10–13]. For example, COP inhibits influenza virus replication by up-regulating the p21 signaling pathway [12]. COP can also reduce renal oxidative damage in diabetic rats by activating Nrf2 signaling pathway [13]. Previous studies have confirmed that COP has anti-tumor properties against a variety of cancers, including gastric cancer [14] and hepatocellular carcinoma [15]. We have reported that COP represses the malignant biological behaviors of bladder cancer cells via repressing the expression of exportin-1 (XPO1) [16]. However, the mechanism of COP against bladder cancer has not been fully explained. Considering natural drugs have the property of multi-target effect, more targets of COP remain to be explored and validated.

Network pharmacology combines system biology and bioinformatics to explain the complex mechanism of drugs, especially natural drugs [17]. Molecular docking predicts the binding patterns and affinity between drugs and their receptors [18]. Molecular dynamics simulation is important for understanding the structure and function of complexes formed by biological macromolecules (such as proteins) and small molecules (such as natural monomers), and can provide guidance for drug design [19]. This study aims to explore the potential targets and molecular mechanisms of COP in bladder bladder cancer through network pharmacology, molecular docking and molecular dynamics simulation, and validate the results of in silico analysis with in vitro assays, and further verify the tumor-suppressive properties of COP with animal models.

## Methods and materials

### Collection of the potential targets of COP and bladder cancer

COP’s targets were collected from the SwissTargetPrediction database, the STITCH database, the TCMSP database, the ETCM database, the symMap database and the CTD database. Using “bladder cancer” as the key word, the targets related to bladder cancer were searched in the GeneCards database, the DisGeNET database, the TTD database and the OMIM database. The genes in the intersection were considered as the targets of COP in bladder cancer treatment.

### Protein-protein interaction (PPI) network

The targets of COP in bladder cancer treatment were imported into the STRING platform, and the condition was set to “Homo sapiens”, and the confidence level was set to medium confidence (0.4) to obtain the PPI network diagram. The generated PPI network was imported into Cytoscape 3.9.0 software, and the cluster analysis was performed by the molecular complex detection (MCODE) plug-in. The parameters were set as follows: degree cutoff = 2, node score cutoff = 0.2, K score = 2 and maximum depth = 100.

### Functional enrichment analysis

Gene ontology (GO) analysis and Kyoto Encyclopedia of Genes and Genomes (KEGG) enrichment analysis were performed using the Metascape database. The default parameter settings were as follows: Minimum overlap = 3, *P* value cutoff < 0.01, minimum enrichment = 1.5. The top 10 GO terms and KEGG pathways with the most gene counts were visualized.

### Identification of the hub targets

The PPI network was evaluated using CytoHubba plug-in, and analyzed based on different algorithms such as betweenness, closeness, degree, maximal clique centrality (MCC), edge percolated component (EPC), maximum neighborhood component (MNC), stress and radiality. The top 20 targets of the results of each algorithm were extracted, and the key target genes were screened with UpSetR. The DriverDBv4 database was used to analyse target gene expression in bladder cancer tissues. The relationship between key target genes and immune infiltration of bladder tumor tissue was analyzed using the TIMER database.

### Construction of the “pathway-hub gene” network

The hub target genes, top 10 KEGG pathways with the most gene counts and crucial proteins in these pathways were imported into the Cytoscape 3.9.0 software to construct the “pathway-hub gene” network. The degree values of the nodes were analyzed by the CytoNCA plug-in to screen out the major hub targets and the key signaling pathways. Pathview package in R was applied to color the targets on critical signaling pathways.

### Molecular docking and molecular dynamics simulation

From the Protein Data Bank database, 3D crystal structures of the major hub targets were downloaded, and the 3D chemical structure of COP was obtained from the PubChem database and saved as SDF format file. SDF files were imported into the ChemBio3D software, to minimize energy states and the files were saved in mol2 format. The AutoDockTools v1.5.7 software was used to preprocess COP and the targets, and the AutoDock Vina v1.1.2 was used for docking. When the binding energy is less than 0 kcal/mol, it indicates that COP may spontaneously bind to the target, and when it is less than − 5 kcal/mol, it indicates that the docking is stable. The PyMOL software was used to visualize the docking results. The Gromacs2022.3 software was used for molecular dynamics simulation. AmberTools22 was used to add GAFF force field to COP, and the Gaussian 16 W software was used for hydrogenation and RESP potential calculation. The simulation environment was set at 300 K temperature and atmospheric pressure (1 Bar), and the force field was selected as Amber99sb-ildn, and the solvent environment was simulated as water molecules (Tip3p water model), and Na + ions were added to neutralize the charge of the system. In the simulation, the steepest descent method was used to minimize the energy of the whole system. Subsequently, 100,000 steps of isothermal isovolumetric ensemble (NVT) equilibrium and isothermal isobaric ensemble (NPT) equilibrium were performed respectively, where the coupling constant was set at 0.1 ps and the equilibrium process lasted for 100 ps. The molecular dynamics simulation process consisted of 5,000,000 steps, each of which is 2 fs long, and the total simulation time reached 100 ns. After the simulation, the trajectory was analyzed and the root mean square deviation (RMSD) and root mean square fluctuation (RMSF) were calculated.

### Cell culture

T24 and BIU-87 cells were available from the Central Laboratory of Renmin Hospital of Wuhan University, and cultured in RPMI-1640 medium (Gibco) with 10% fetal bovine serum (HyClone), 100 U/mL penicillin, and 100 µg/mL streptomycin (Gibco) at 37 °C in 5% CO_2_. COP (Aladdin Bio-Chem Technology Co. Ltd.; Purity > 98%) were dissolved in dimethyl sulfoxide (DMSO; Beyotime) and diluted with the medium for subsequent experiments.

### Western blot

The bladder cancer cell lines were treated with different doses of COP (10, 20, 40 µmol/L) for 24 h. After that, pre-cooled RIPA lysis buffer (Thermofisher) was used to extract the total protein. The protein samples (20 µg/lane) were separated by sodium dodecyl sulfate polyacrylamide gel electrophoresis, and then electrically transferred onto polyvinylidene fluoride membranes. Subsequently, the membranes were cut based on the ladder, according to the predicted molecular weights of the proteins. After blocking with 5% skimmed milk, the membranes were incubated with the primary antibodies (1: 1000), and secondary antibody (1: 5000) (Abcam), respectively. Finally, the protein bands were visualized using an enhanced chemiluminescence kit (Biozym) and quantitatively analyzed using Image Lab™ software.

### Animal experiments

All animal procedures were approved by the Animal Research Ethics Review Committee of Renmin Hospital of Wuhan University (Approval No. WRDM20241204D). A total of 48 6-week-old male nude mice (BALB/C, Model Animal Center of Wuhan University) were used. The mice were kept in specific pathogen free (SPF) condition with free access to food and water. In tumorigenesis assay and metastasis assay, the nude mice were randomly divided into three groups [blank control group, low-dose COP treatment group (10 µmol/L) and high-dose COP treatment group (40 µmol/L), 8 mice in each group]. In tumorigenesis assay, after treatment, T24 cell suspension was prepared with phosphate buffer saline (PBS), and the cell density was adjusted to 1 × 10^7^ cells/ml. Then 0.1 mL cell suspension was injected into the subcutaneous tissue of the back of each mouse through a sterile syringe. Tumor growth was monitored daily after cell injection. By measuring the length and width of the tumor, the tumor volume was calculated using the formula V = 1/2 x length x width. The nude mice were euthanized 30 days after inoculation, and the tumors were isolated and weighted. For the metastasis model, 0.1 mL cell suspension was injected into the tail vein of nude mice. After 21 days of continuous feeding, the mice were sacrificed with cervical dislocation, and the lungs and livers of each mouse were taken. Lung and liver tissues were fixed, paraffin-embedded, sectioned, and stained with hematoxylin-eosin (H&E) staining solution. Finally, the metastatic nodules in lung and liver were observed under microscope.

### Statistical analysis

SPSS 20.0 was used for statistical analyses. The normality of the data was tested with Shapiro-Wilk test, and the homogeneity of variances was tested with Levene’s test. All data were expressed as “mean ± standard deviation” for the in vitro and in vivo assays. The comparison between different groups was performed with one-way ANOVA and Tukey *post-hoc* test. *P* < 0.05 is indicative of statistically significant difference.

## Results

### Identification of cop’s targets and bladder cancer-related targets

**Fig. 1:**
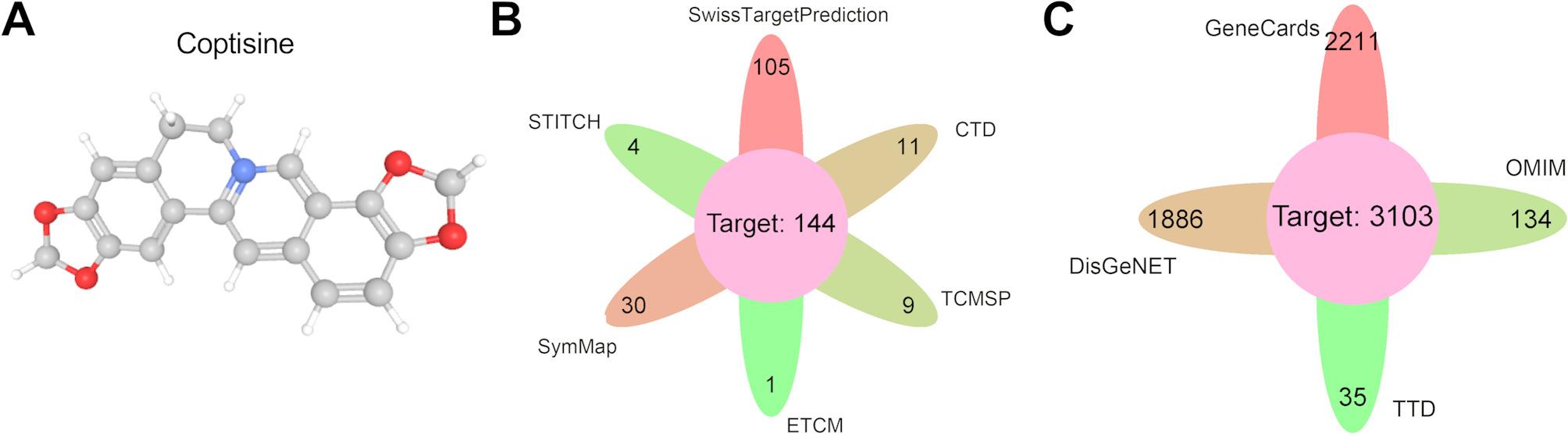
Collection of the targets of COP and bladder cancer-related genes. A. 3D chemical structure of COP B. Statistical graph of COP’s targets in the SwissTargetPrediction, STITCH, SymMap, ETCM, TCMSP, CTD databases C. Statistical graph of bladder cancer-related targets in GeneCards, DisGeNET, TTD and OMIM databases

The 3D chemical structure of COP is shown (Fig. 1A). A total of 105 targets were obtained from the SwissTargetPrediction database, and 4 action targets were found in the STITCH database, and 30 targets were provided by the SymMap database. 1, 9 and 11 COP-related targets were identified from ETCM, TCMSP and CTD databases, respectively (Fig. 1B). A total of 144 targets were obtained by combining the targets of these 6 databases and removing the duplicate ones. In addition, using “bladder cancer” as the keyword, 2211, 1886, 35 and 134 bladder cancer-related targets were obtained from GeneCards, DisGeNET, TTD and OMIM databases, respectively (Fig. 1C). After merging and removing duplicate targets, 3103 disease targets were obtained.

### Identification of the targets of COP in bladder cancer treatment

**Fig. 2:**
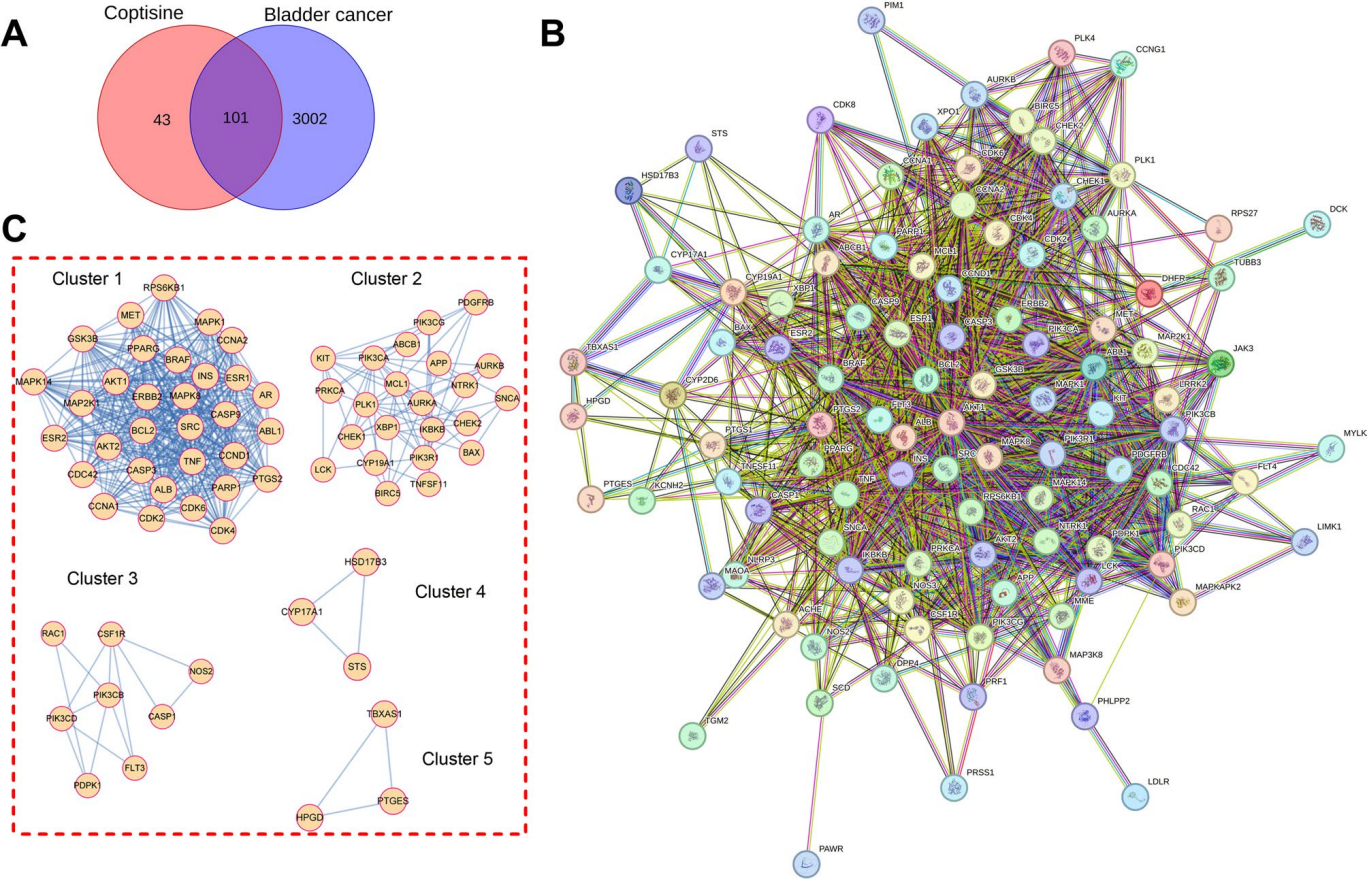
Construction and analysis of the PPI network of COP’s targets in bladder cancer treatment.**A** The Venn diagram showing the targets in the intersection of COP’s targets and bladder cancer-related targets **B** The PPI network of COP’s targets in bladder cancer treatment. Nodes represent proteins, and edges represent protein-protein interactions **C**. The MCODE plug-in was applied to filter important clusters of PPI networks

**Fig. 3:**
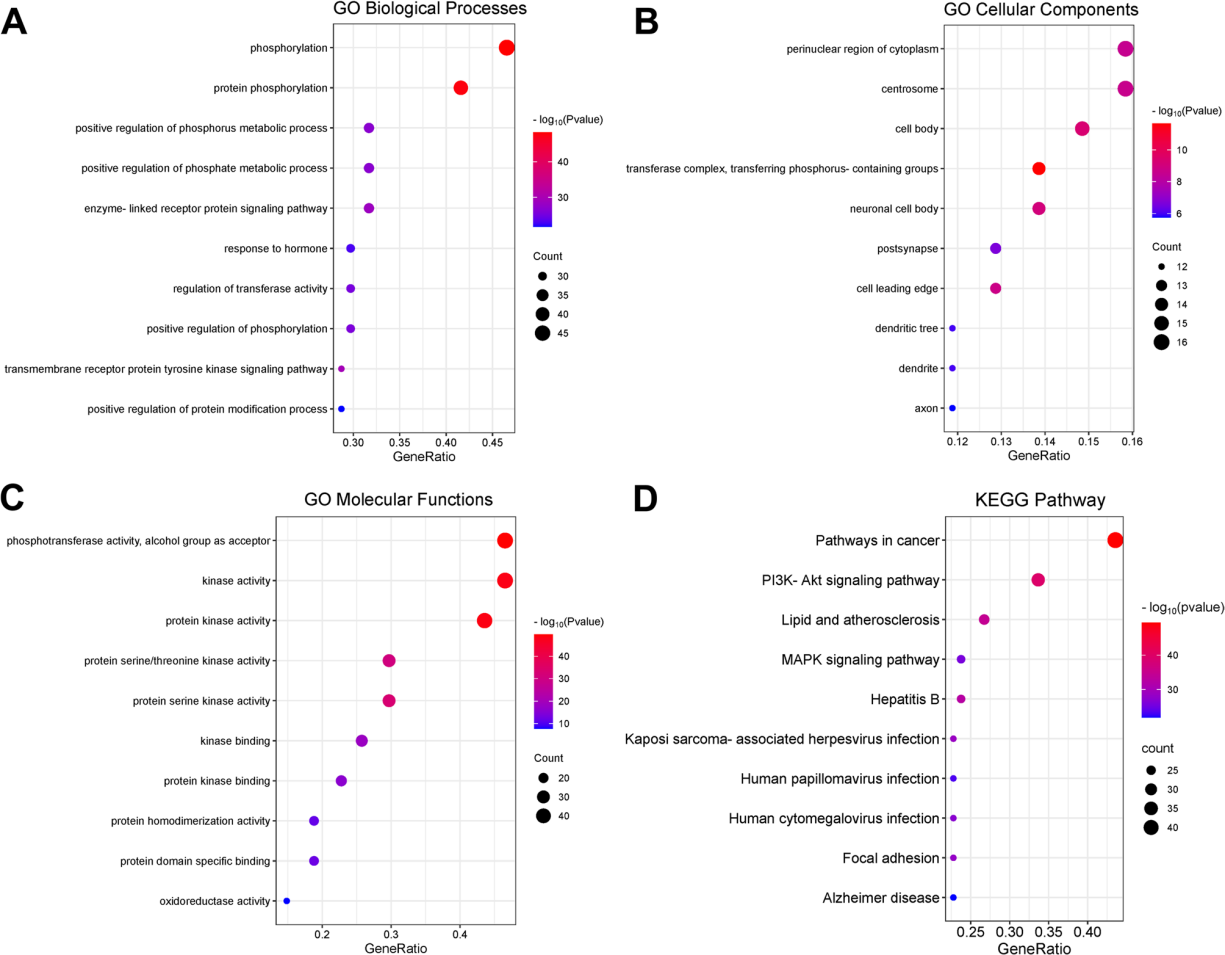
GO and KEGG enrichment analyses of COP’s targets in bladder cancer treatment.**A**-**D**. Enrichment bubble map of the top 10 terms of biological processes (**A**), cell components (**B**), molecular functions (**C**), and KEGG pathway (**D**) associated with COP’s targets in bladder cancer treatment. The bubble size represents the gene count, and the bubble color represents the *P*-value

The 144 drug targets and 3103 disease-related targets were analyzed with a Venn diagram, and a total of 101 targets in the intersection were obtained, which were regarded as the targets of COP in bladder cancer treatment (Fig. 2A). The 101 targets were imported into the STRING database to construct a PPI network, and a network of 101 nodes and 1338 edges was obtained (Fig. 2B). With MCODE plug-in analysis, 5 gene clusters were obtained, and among them, the cluster 1 had the highest score, which consisted of 32 nodes and 408 edges (Fig. 2C; Table 1). Additionally, the 101 targets were imported into the Metascape database for GO and KEGG pathway enrichment analyses. The results of GO analysis showed that 320 biological processes (BP), 86 cell components (CC) and 85 molecular functions (MF) were associated with the targets. The top 10 BP, CC, and MF terms with the most gene counts were then selected for visualization. The top 5 terms of BP are phosphorylation, protein phosphorylation, enzyme-linked receptor protein signaling pathway and positive regulation of phosphorus metabolic process, positive regulation of phosphate metabolic process (Fig. 3A). The top 5 terms in CC were centrosome, perinuclear region of cytoplasm, cell body, transferase complex, transferring phosphorus-containing groups and neuronal cell body (Fig. 3B). The top 5 terms in MF were phosphotransferase activity, alcohol group as acceptor, kinase activity, protein kinase activity, protein serine kinase activity, protein serine/threonine kinase activity (Fig. 3C). In addition, KEGG enrichment analysis showed that a total of 165 pathways were associated with the 101 targets. Among the top 10 pathways, pathways in cancer (hsa05200), PI3K-Akt signaling pathway (hsa04151), focal adhesion (hsa04510) were associated with cancer biology (Fig. 3D).

**Table 1:** Clusters of genes of Coptisine against bladder cancer

Cluster	Score (Density*#Nodes)	Nodes	Edges	Node IDs
1	26.323	32	408	ESR1, CASP3, CDC42, TNF, ALB, CCND1, CCNA2, CDK4, ESR2, AKT2, BCL2, ERBB2, SRC, MAPK14, AR, ABL1, RPS6KB1, PTGS2, MAPK8, MAPK1, PARP1, CCNA1, PPARG, CASP9, INS, MAP2K1, AKT1, GSK3B, BRAF, CDK2, CDK6, MET
2	5.909	23	65	CHEK1, XBP1, MCL1, SNCA, PIK3CG, PIK3CA, BAX, AURKA, PDGFRB, CYP19A1, ABCB1, LCK, KIT, PLK1, APP, CHEK2, BIRC5, NTRK1, IKBKB, AURKB, PIK3R1, PRKCA, TNFSF11
3	3.714	8	13	NOS2, CASP1, FLT3, PDPK1, CSF1R, PIK3CB, RAC1, PIK3CD
4	3	3	3	HSD17B3, CYP17A1, STS
5	3	3	3	HPGD, TBXAS1, PTGES

### Identification of the hub targets among cop’s targets in bladder cancer treatment

**Fig. 4:**
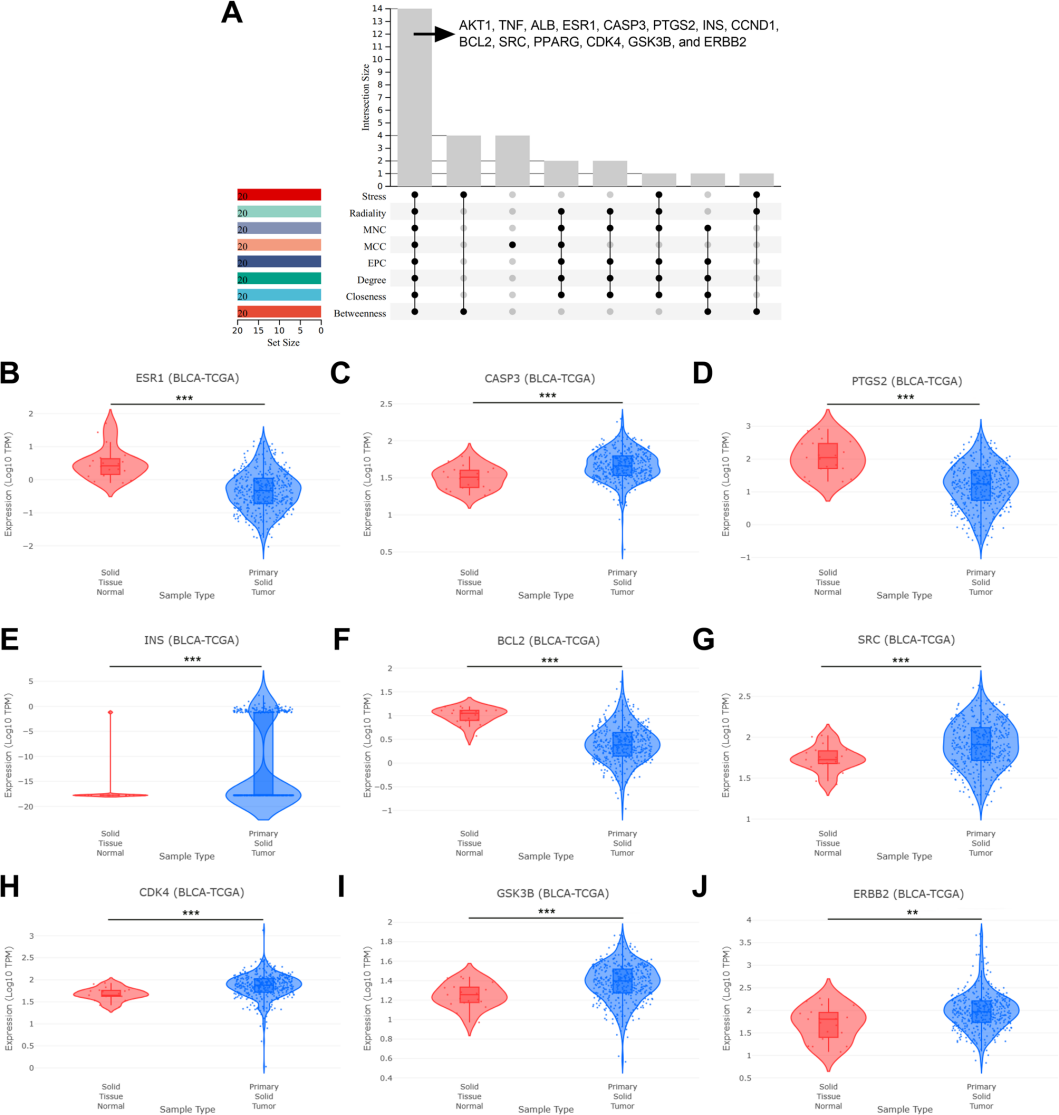
Identification of the hub targets of COP in bladder cancer treatment.**A** UpSetR was applied to extract the intersection of the results of analyes of betweenness, closeness, degree, MCC, EPC, MNC, stress and radiality B-J. Expression levels of ESR1 (**B**), CASP3 (**C**), PTGS2 (**D**), INS (**E**), BCL2 (**F**), SRC (**G**), CDK4 (**H**), GSK3B (**I**) and ERBB2 (**J**) in normal tissues and bladder cancer tissues were compared, respectively. Red represents normal tissue and blue represents cancerous tissue **P < 0.01, ***P < 0.001

In order to determine the hub targets from the 101 targets, the top 20 genes in PPI network were further screened by CytoHubba plugin (Table 2). Subsequently, the results of different algorithms were cross-analyzed, and 14 genes in the intersection were obtained, which were AKT1, TNF, ALB, ESR1, CASP3, PTGS2, INS, CCND1, BCL2, SRC, PPARG, CDK4, GSK3B, and ERBB2 (Fig. 4A). In addition, we analyzed the expression of these genes in bladder cancer using the DriverDBv4 database (Fig. 4B-J). The results showed that compared with normal tissues, ESR1, PTGS2 and BCL2 in bladder cancer tissues were significantly down-regulated; CASP3, INS, SRC, CDK4, GSK3B and ERBB2 were significantly up-regulated.

**Table 2:** The top 20 gene rank in CytoHubba plug-in

Betweenness	Closeness	Degree	EPC	MCC	MNC	Stress	Radiality
AKT1	AKT1	AKT1	AKT1	BCL2	AKT1	AKT1	AKT1
TNF	TNF	TNF	CASP3	CASP3	BCL2	TNF	TNF
ALB	BCL2	BCL2	INS	ESR1	TNF	ALB	BCL2
ESR1	CASP3	CASP3	BCL2	CCND1	CASP3	ESR1	CASP3
CASP3	ALB	ALB	ESR1	AKT1	ALB	CASP3	ALB
PTGS2	ESR1	ESR1	ALB	SRC	ESR1	BCL2	ESR1
INS	INS	INS	TNF	PARP1	INS	PTGS2	INS
CCND1	SRC	SRC	SRC	ALB	SRC	INS	SRC
BCL2	CCND1	CCND1	CCND1	TNF	CCND1	CCND1	CCND1
SRC	PTGS2	GSK3B	PPARG	CASP9	GSK3B	SRC	PTGS2
APP	GSK3B	PTGS2	PIK3CA	PTGS2	PTGS2	PPARG	GSK3B
PPARG	PIK3CA	PIK3CA	GSK3B	MAP2K1	PIK3CA	CDK4	PPARG
DHFR	PPARG	PPARG	PTGS2	GSK3B	PPARG	CDK2	PIK3CA
CDC42	ERBB2	ERBB2	PARP1	MAPK14	ERBB2	APP	PARP1
CDK2	PARP1	PARP1	ERBB2	BRAF	PARP1	CYP19A1	ERBB2
CDK4	CDK4	CDK4	MAPK8	INS	CDK4	GSK3B	CDK4
CYP19A1	CASP9	CASP9	ABL1	ERBB2	CASP9	CDC42	CASP9
ABL1	MCL1	MCL1	CASP9	CDK4	MCL1	PIK3CA	MCL1
GSK3B	MAPK8	ABL1	CDK4	PPARG	ABL1	DHFR	MAPK8
ERBB2	ABL1	MAPK8	MCL1	MAPK1	MAPK8	ERBB2	CDK2

### Construction and analysis of “pathway-hub gene” network

**Fig. 5:**
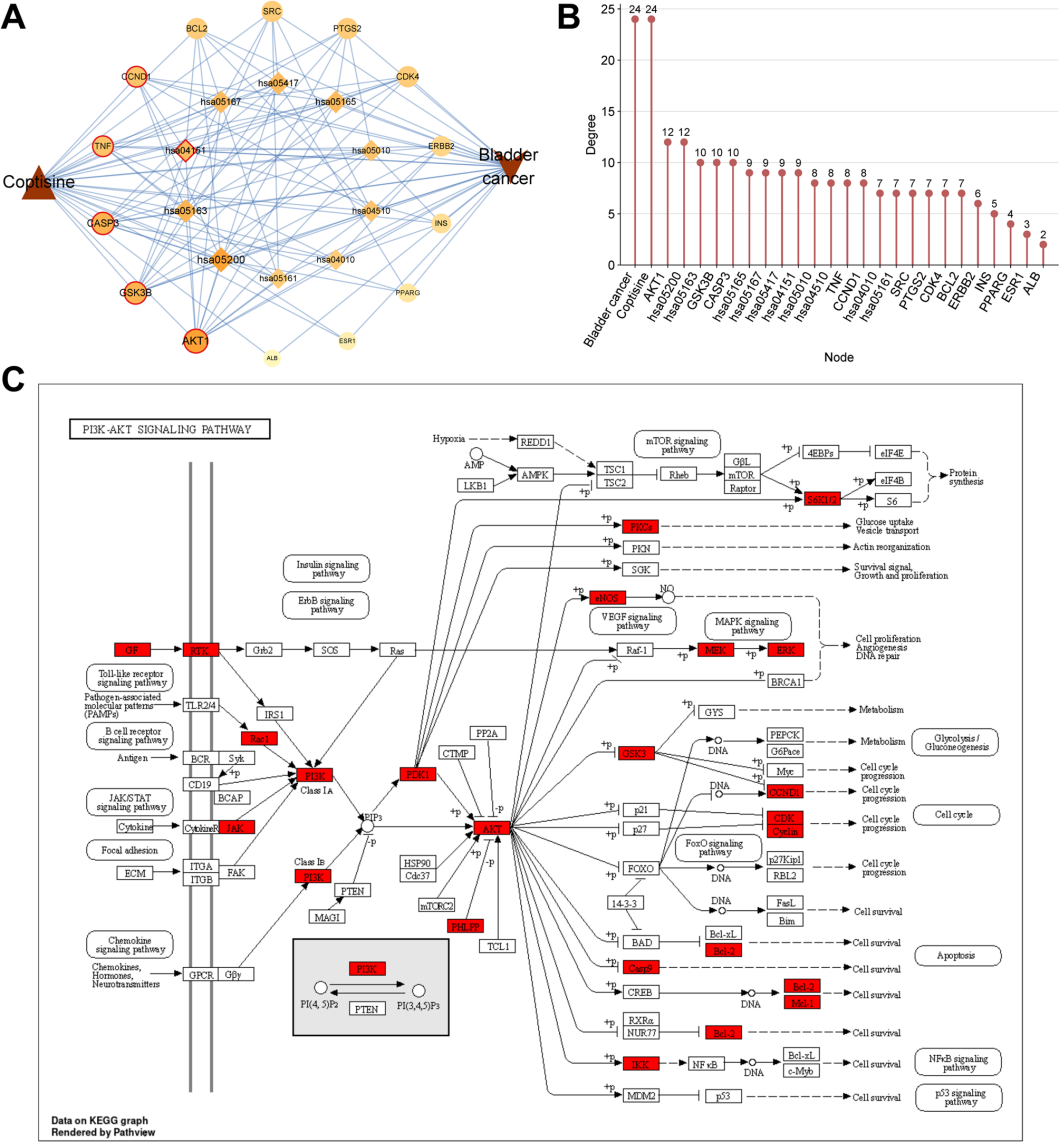
Construction and analysis of “pathway-hub gene” network**A**. “Pathway-Hub gene network” was constructed using Cytoscape 3.9.0 software. The larger the degree value, the larger the node and the darker the color **B**. Statistical diagram of the degree value of each node in the network **C**. R package “Pathview” was used to map the targets of COP in the PI3K/Akt pathway

**Fig. 6:**
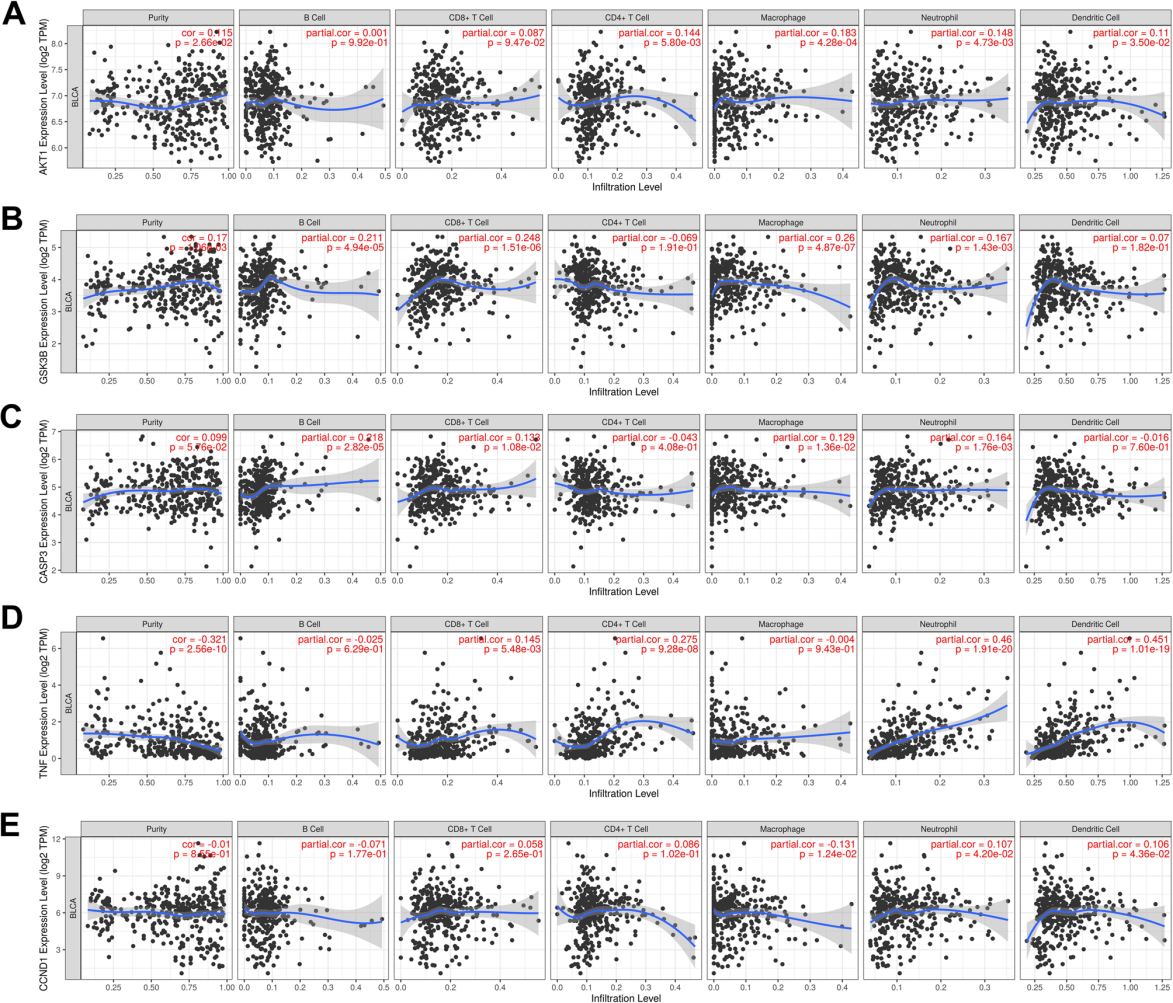
Correlation between major hub genes and immune cell infiltration in bladder cancer tissues. A-E. TIMER database was used to analyze the correlation between major hub targets (AKT1, CASP3, GSK3B, TNF, CCND1) and infiltration levels of different immune cells in bladder cancer

In order to further explore the key targets and pathways associated with COP in bladder cancer, the 14 hub genes, the top 10 KEGG pathways with the most gene counts and crucial regulators in these pathways were imported into Cytoscape 3.9.0 software to obtain a “pathway-hub gene” network, comprising 26 nodes and 116 edges (Fig. 5A). CytoNCA plug-in analysis showed that the PI3K-Akt signaling pathway (degree = 9) may be the key pathway in the treatment of bladder cancer by COP (Fig. 5B), and AKT1 (degree = 12), GSK3B (degree = 10), CASP3 (degree = 10), TNF (degree = 8) and CCND1 (degree = 8) were considered to be the main hub targets (Table 3). The targets in the PI3K/AKT pathway were shown using KEGG PathView, and the targets of COP were marked in red (Fig. 5C). Additionally, the relationship between the expression level of major hub targets and immune cell infiltration was analyzed using TIMER database. The results showed that the expression level of AKT1 in bladder cancer was positively correlated with tumor purity, infiltration abundance of CD4 + T Cell, Macrophage, Neutrophil and Dendritic Cell (Fig. 6A). The expression of GSK3B was positively correlated with tumor purity, infiltration abundance of B Cell, CD8 + T Cell, Macrophage and Neutrophil (Fig. 6B). The expression level of CASP3 was positively correlated with infiltration abundance of B Cell, CD8 + T Cell, Macrophage and Neutrophil (Fig. 6C). The expression level of TNF was positively correlated with the infiltration abundance of CD8 + T Cell, CD4 + T Cell, Neutrophil, and Dendritic Cell, but negatively correlated with tumor purity (Fig. 6D). The expression level of CCND1 was positively correlated with the infiltrate abundance of Neutrophil and Dendritic Cell, but negatively correlated with that of Macrophage (Fig. 6E). These results suggested that the major hub targets participated in regulating the immune environment of bladder cancer tissues.

**Table 3:** Summary of 5 main hub genes

Target	UniProt ID	Betweenness	Closeness	Degree	EPC	MCC	MNC	Stress	Radiality
AKT1	P31749	735.0915184	87.33333333	75	29.375	7,308,734,417,246,260	75	6434	3.79
GSK3B	P49841	131.5990642	76.33333333	53	26.568	7,248,988,878,034,560	53	2140	3.57
CASP3	P42574	389.1077871	83.83333333	68	29.258	7,309,116,200,273,020	68	4560	3.72
TNF	P01375	708.8829089	84.33333333	69	28.284	7,276,517,174,494,270	68	5880	3.73
CCND1	P24385	325.141379	77.66666667	56	27.327	7,308,915,398,432,660	56	3712	3.59

### The binding affinities between COP and the major hub targets

**Fig. 7:**
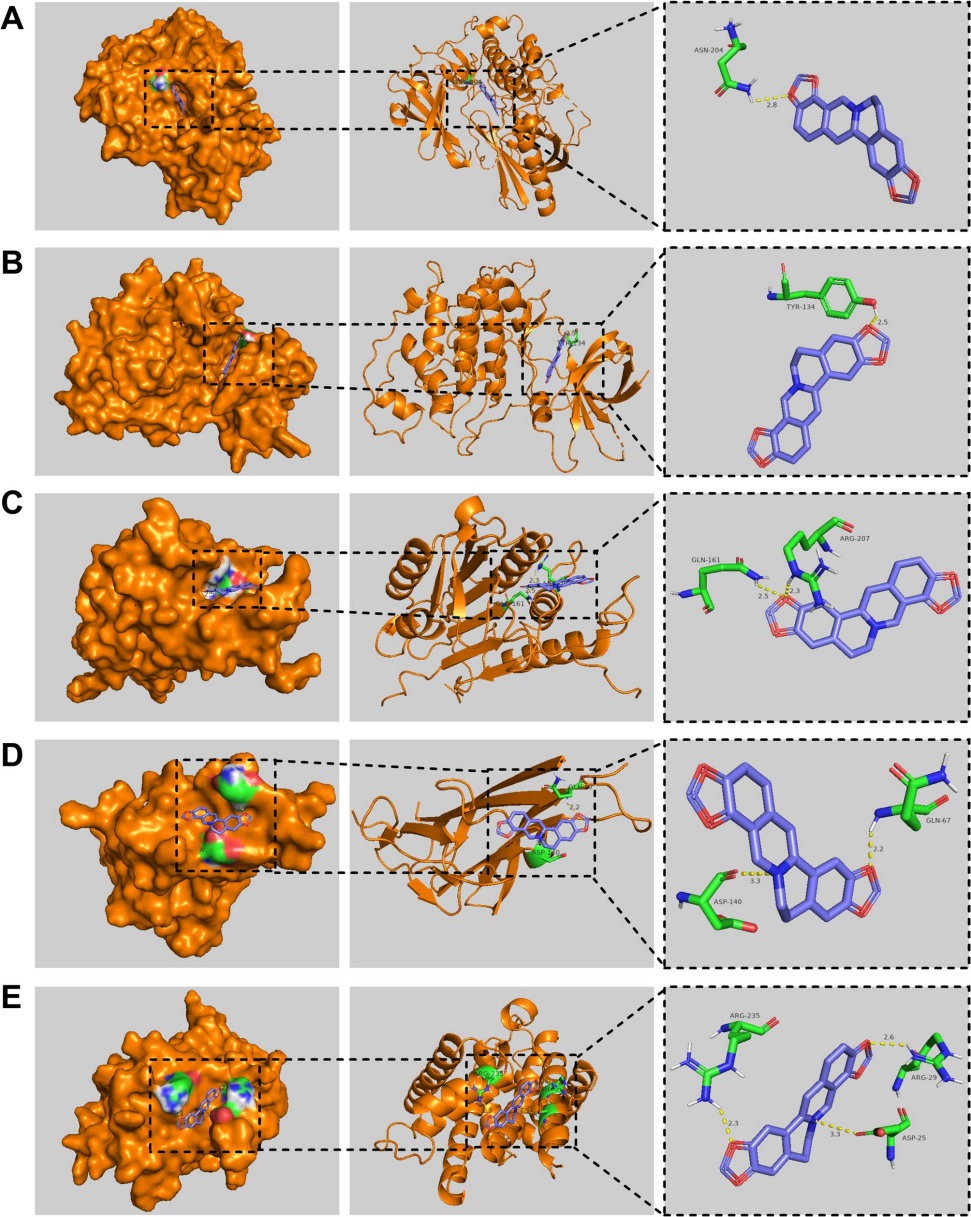
Molecular docking of COP and major hub targets. E. Molecular docking 3D diagram of COP with AKT1 (**A**), GSK3B (**B**), CASP3 (**C**), TNF (**D**), CCND1 (**E**). Blue represents COP, green represents amino acid residues surrounding the binding bag, yellow dashed lines represent hydrogen bonds, and orange represents the targets

**Fig. 8:**
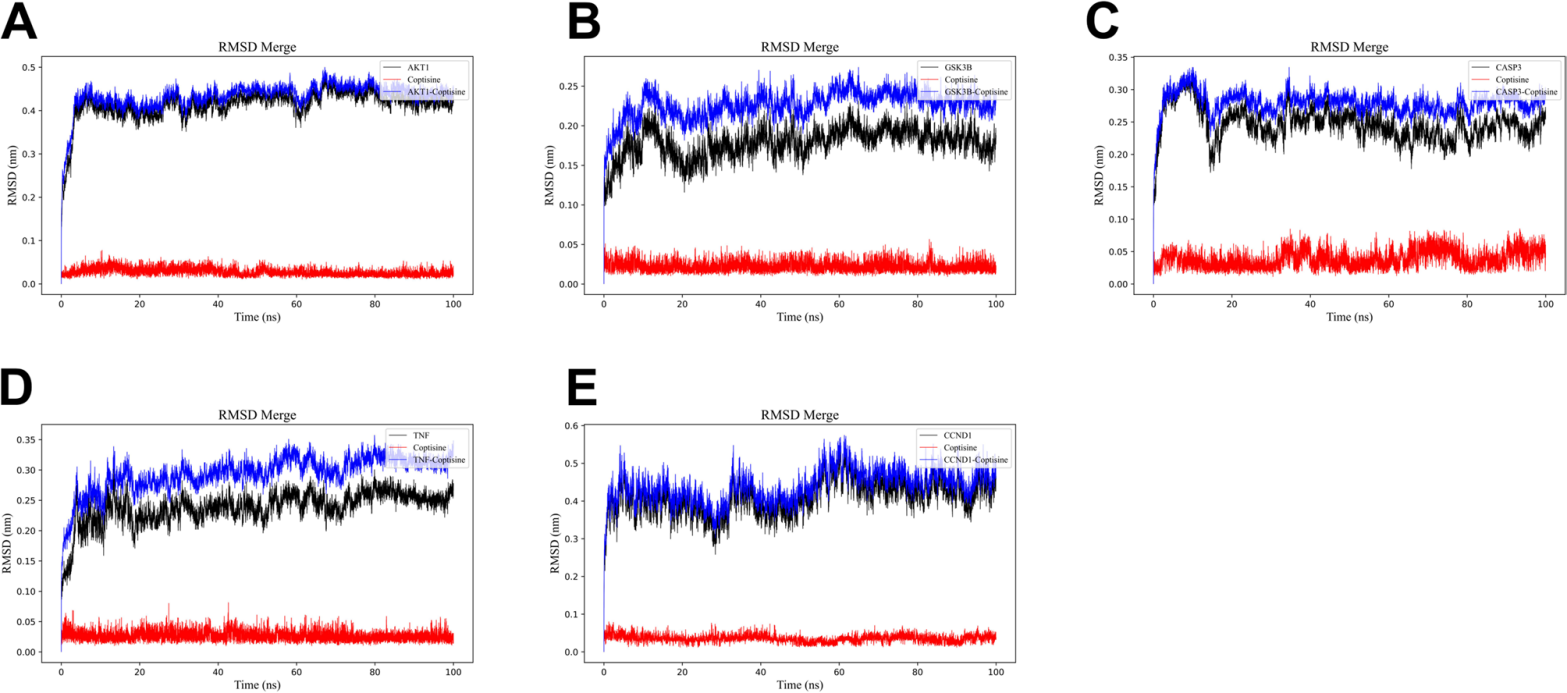
RMSD diagram of molecular dynamics simulation. E. RMSD diagram of the complexes formed by COP with AKT1 (**A)**, GSK3B (**B**), CASP3 (**C**), TNF (**D**), CCND1 (**E**). Red indicates COP fluctuations, black indicates protein target fluctuations, and blue indicates complex fluctuations. The horizontal axis is the simulation time, and the longitudinal axis is RMSD

**Fig. 9:**
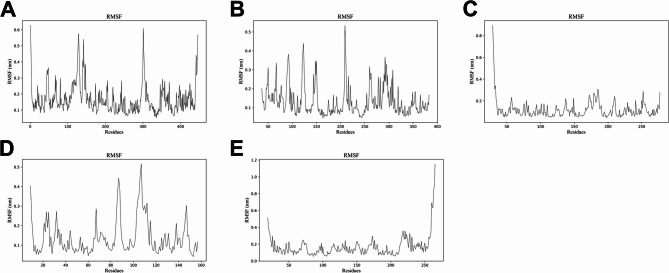
RMSF diagram of molecular dynamics simulation. E. RMSF of the complexes formed by COP with AKT1 (**A**), GSK3B (**B**), CASP3 (**C**), TNF (**D**), CCND1 (**E**). The horizontal coordinate is the amino acid residue position, and the vertical coordinate is RMSF

Next, molecular docking was performed to evaluate the possibility of the binding between COP and major hub targets. The results showed that COP could form a complex stably with AKT1, GSK3B, CASP3, TNF and CCND1, with a binding energy of − 11.0, − 9.1, − 8.1, − 7.3 and − 7.4 kcal/mol, respectively (Fig. 7A-E). In general, the binding energies of coptisine with AKT1, GSK3B, CASP3, TNF and CCND1 were all less than − 7 kcal/mol, indicating that COP could spontaneously and stably bind to the major hub targets. To validate the results of molecular docking, molecular dynamics simulation was performed. RMSD is an index to evaluate the change of protein structure. The results showed that the RMSD values of the complexes formed by AK1, GSK3B, CASP3, TNF and CCNE1 and COP were large after the simulation began, and then quickly stabilized, and the time to reach the stable state was about 4 ns, 15 ns, 3 ns, 15 ns and 3 ns, respectively. On the whole, after the equilibrium was reached, the fluctuation range of RMSD value of each complex remained within a small range, about 0.06 nm, 0.05 nm, 0.1 nm, 0.13 nm and 0.25 nm, respectively (Fig. 8A-E). The results indicated that AK1, GSK3B, CASP3, TNF and CCNE1 were stable in binding with COP. RMSF is an index to evaluate the flexibility of amino acid residues. The results showed that the RMSF values of the complexes formed by AK1, GSK3B, CASP3, TNF and CCND1 and COP were smaller in the binding part, but larger in the unbinding part (Fig. 9A-E), indicating that COP binding had a certain impact on the stability of AK1, GSK3B, CASP3, TNF, and CCNE1. Generally, these data suggested that COP could bind stably with AK1, GSK3B, CASP3, TNF and CCND1, probably functioned as an inhibitor of these proteins.

### COP inactivates PI3K/AKT pathway in bladder cancer cells

**Fig. 10:**
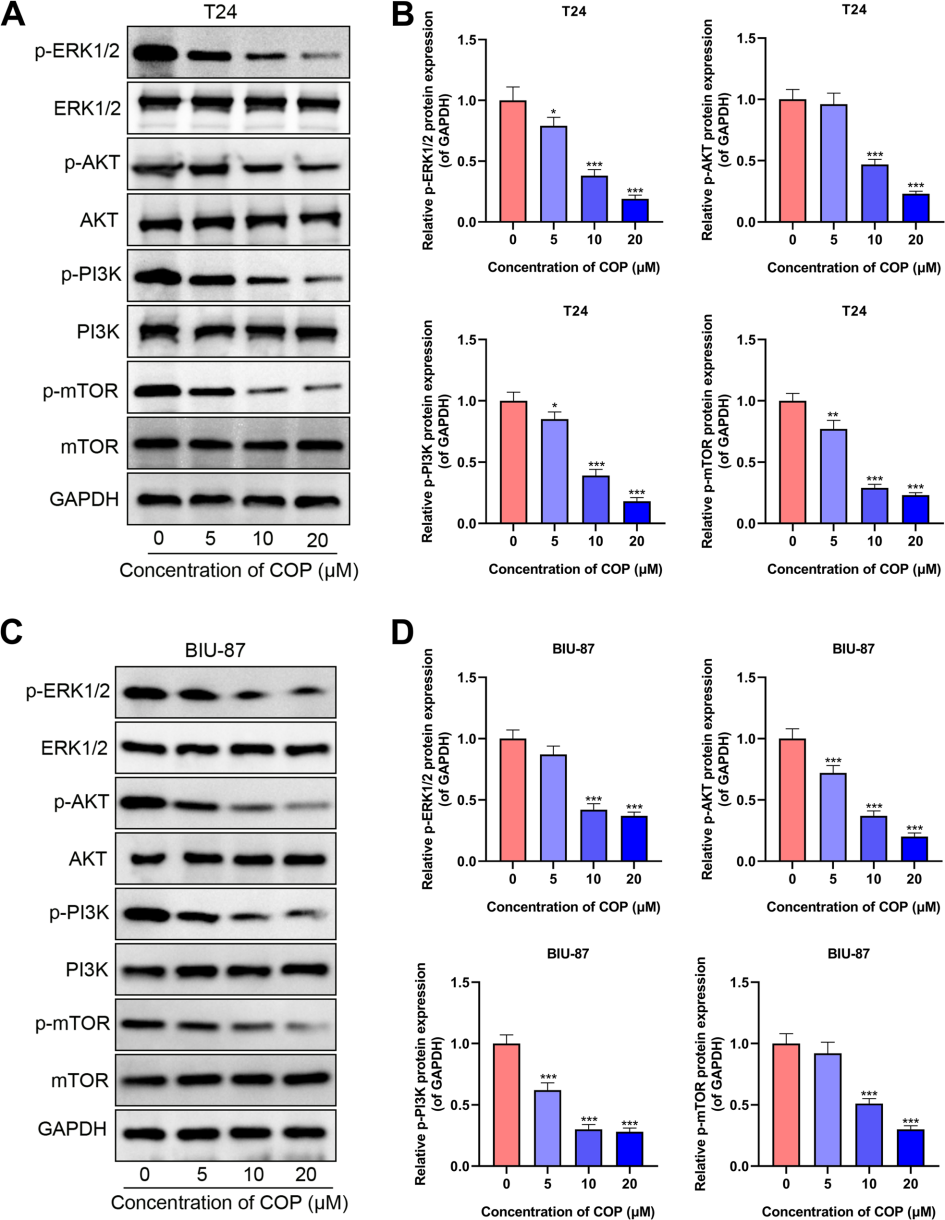
COP inhibits PI3K/AKT/mTOR pathway activation in bladder cancer cells. E. The expression levels of p-ERK1/2, p-AKT, p-PI3K and p-mTOR proteins in T24 cells (**A**&**B**) and BIU-87 cells (**C**&**D**) were detected by Western blot after 24 h treatment with different concentrations of COP (0, 10, 20, 40 µmol/L) *P < 0.05, **P < 0.01, ***P < 0.001

Since the analysis of “pathway-hub gene” network suggested that the PI3K/AKT signaling pathway was a key signaling pathway affected by COP in bladder cancer treatment, the effects of COP on the activity of PI3K/AKT signaling pathway were further explored. After T24 cells and BIU-87 cells were treated with different concentrations of COP (0, 10, 20, 40 µmol/L) for 24 h, the phosphorylated protein expression levels and total protein expression levels of ERK1/2, AKT, PI3K p85, mTOR were detected by Western blot. The results showed that phosphorylation of ERK1/2, AKT, PI3K p85, and mTOR was significantly inhibited by COP in a dose-dependent manner (Fig. 10A-D). These data further validated that COP could inhibit the activation of PI3K/AKT pathway and affect signal transduction of downstream pathways such as MAPK and mTOR pathways.

### COP repressed the tumorigenesis and metastasis of bladder cancer cells in vivo

**Fig. 11:**
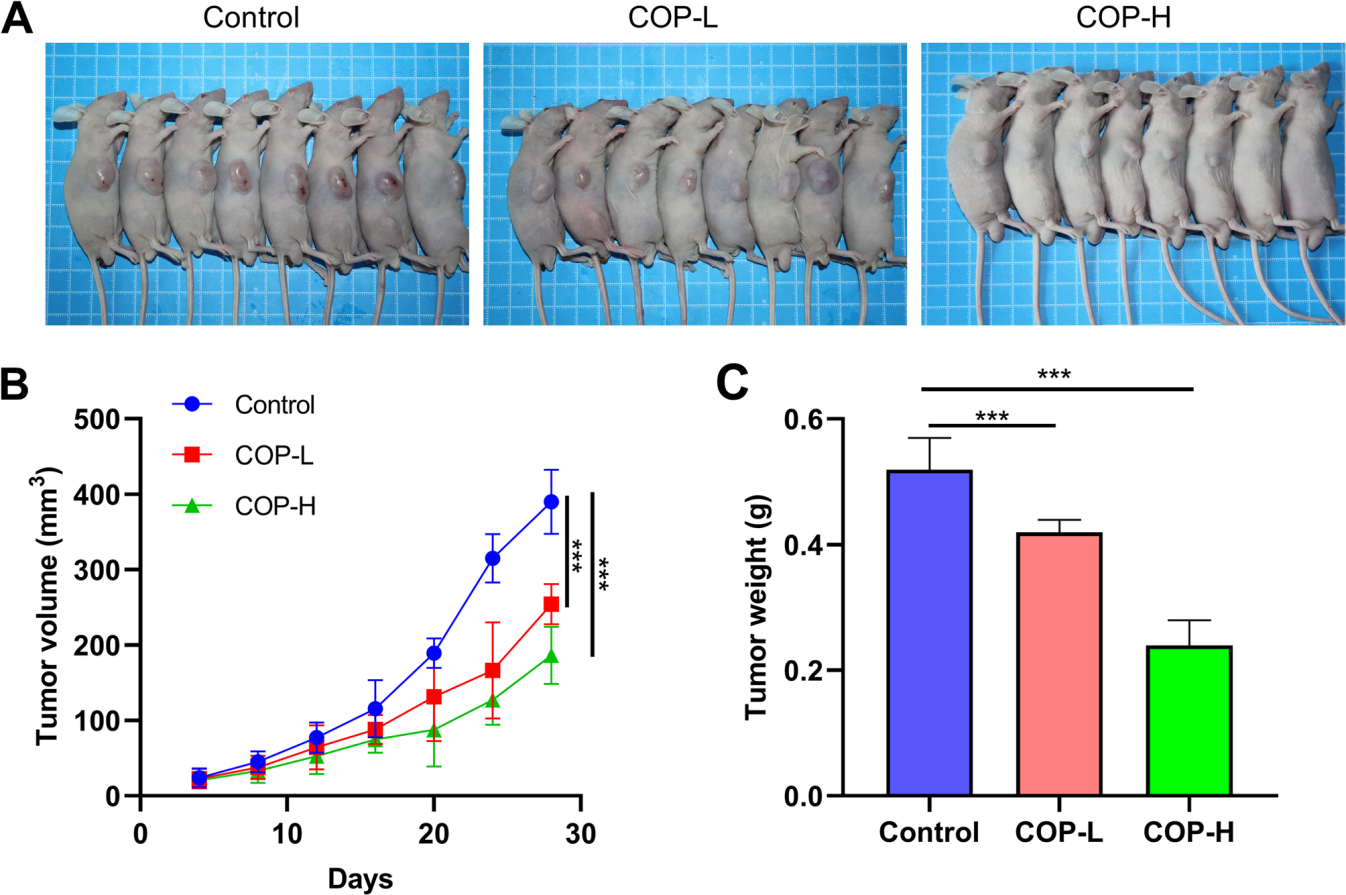
COP represses tumorigenesis of bladder cancer cell line T24 in nude mice. C. After the treatment of T24 cells with 10 µmol/L COP (COP-L) or 40 µmol/L COP (COP-H) for 24 h, the cells were inoculated into the subcutaneous tissues of nude mice, and T24 cells, which were not treated with COP were used as the control. The the volume (**A**, **B**) and weight (**C**) of the tumor were compared ***P < 0.001

**Fig. 12:**
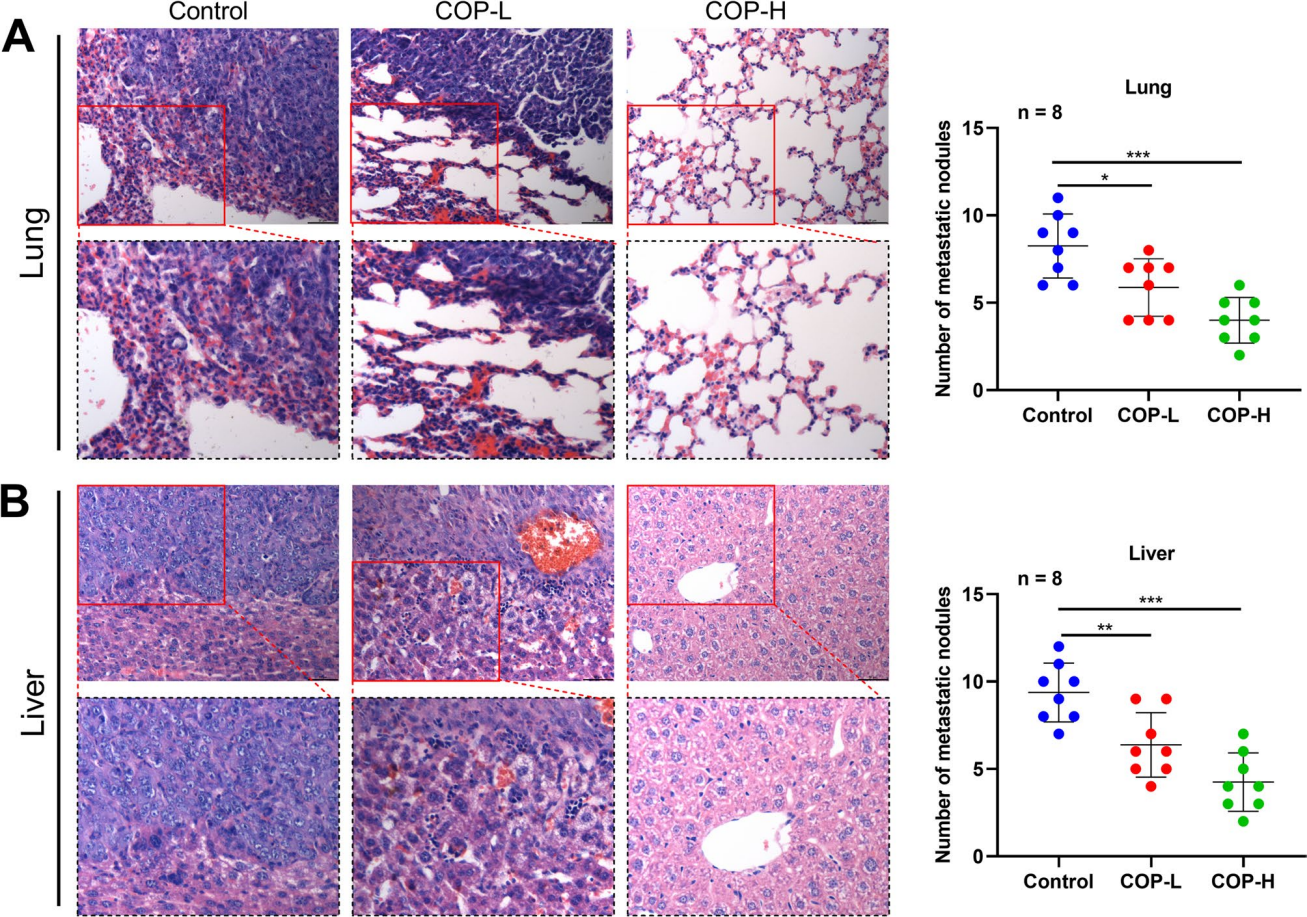
COP inhibits metastatic ability of bladder cancer cell line T24. C. T24 cells treated with 10 µmol/L COP (COP-L) or 40 µmol/L COP (COP-H) for 24 h were injected into nude mice through the tail vein, and T24 cells, which were not treated with COP were used as the control. The number of metastatic nodules in lung (**A**) and liver (**B**) in each group were compared *P < 0.05, **P < 0.01, **P < 0.001

In order to further verify the regulatory effect of COP on the malignant phenotype of bladder cancer cells, T24 cells treated different doses of COP (control group, low-dose group COP = L and high-dose group COP-H), were injected subcutaneously into the mice. As shown, the graft volume in the COP treatment group was significantly lower than that in the control group, and the tumor inhibition effect in the high-dose group was more significant (Fig. 11A**&B**). Correspondingly, the weight of tumor in COP treatment groups was also significantly lower than that in the control group, and the tumor inhibition effect in the high-dose group was more significant (Fig. 11C). T24 cells were also injected them into nude mice through the tail vein. As shown, the number of metastatic nodules formed in the lung of nude mice in the COP treatment groups was significantly less than that in the control group, and the tumor inhibition effect was more significant in the high-dose group (Fig. 12A). Correspondingly, the number of metastatic nodules formed by T24 cells in the liver of nude mice was also significantly lower in COP treatment groups than that in the control group, and the tumor inhibition effect was more significant in the high-dose group (Fig. 12B).

## Discussion

We previously reported that COP represses the malignant biological behaviors of bladder carcinoma cells and regulates XPO1 expression [16]. In this study, through network pharmacology, we identified the key downstream signaling pathway PI3K/AKT through which COP exerts anti-bladder cancer effects. In addition, through molecular docking and molecular dynamics simulation, this study identified a series of potential molecular targets of COP, including AK1, GSK3B, CASP3, TNF and CCNE1. Finally, we used in vivo and in vitro models to further verify the anti-cancer properties of COP, which further supports and expands the findings of our previous study [16]. To our best knowledge, this is the first work which comprehensively investigates the molecular mechanisms of COP in repressing the progression of bladder cancer.

In recent years, network pharmacology has become a popular tool for determining the pharmacological effects of drugs on diseases. For example, with network pharmacology, it has been found that osthole can inhibit bladder cancer cell migration, invasion, and epithelial-mesenchymal transition by regulating PI3K/AKT and JAK/STAT3 signaling pathways [20]. With network pharmacology and in vitro assays, it is reported that galangin can induce apoptosis of bladder cancer cells by activating p53 signaling pathway [21]. In this study, through network pharmacology, it was demonstrated that AKT1, GSK3B, CASP3, TNF, and CCND1 may be the crucial targets of COP in bladder cancer treatment. AKT1 is a serine/threonine protein kinase, which plays a crucial role in various biological processes such as glucose metabolism, cell proliferation, migration and apoptosis [22]. AKT1 is highly expressed in bladder cancer and is associated with its malignant progression [23]. Nuclear accumulation of GSK3B is associated with progression and poor survival of bladder cancer patients; GSK3B knockdown inhibits the viability of bladder cancers [24]. CASP3 is a key enzyme in the apoptosis pathway and a major executor of apoptosis [25]. TNF is an important inflammatory mediator, which can promote the release of pro-inflammatory factors and also plays an important role in the occurrence and development of bladder cancer [26]. CCND1 is a highly conserved cyclin, and its amplification is a risk factor for bladder cancer metastasis [27]. Notably, this study found that the expression levels of AKT1, GSK3B, CASP3, TNF, and CCND1 were associated with immune cell infiltration in bladder cancer, suggesting that these targets could be used as potential predictors of bladder cancer immunotherapy. In addition, molecular docking and molecular dynamics simulations showed strong binding affinities between COP and these major hub targets. This suggests that COP may act as a natural inhibitor against these targets and thus play a promising role in the treatment of bladder cancer.

The PI3K/AKT signaling pathway plays an important role in regulating various cell biological processes [28]. In bladder cancer, up to 40% of tumors exhibit constitutive activation of the PI3K/AKT/mTOR pathway [29]. Activation of PI3K/AKT signaling pathway is associated with tumorigenesis, progression, metastasis and cisplatin resistance of bladder cancer [30–32]. It has been reported that gypenosides, cryptotanshinone, and deoxyschisandrin inhibit the progression of bladder cancer by modulating PI3K/AKT pathway [33–35]. This suggests that targeting the PI3K/AKT signaling pathway may be a potential strategy for bladder cancer treatment. The in silico analysis of this study also suggested that PI3K/AKT pathway may be a key pathway in the mechanism of COP in bladder cancer treatment, which is consistent with the mechanism of COP in treating other human malignancies [15]. As expected, after T24 cells and BIU-87 cells were treated with COP, phosphorylated ERK1/2, AKT1, and PI3K p85 levels were significantly reduced. Considering that in the present work, AKT1 was identified as the target of COP, it is reasonable to suppose that COP represses the activation of PI3K/AKT signaling and the related malignant behaviors of bladder cancer cells via directly suppressing AKT1. However, whether COP may inhibit the PI3K/AKT signaling pathway through other mechanisms needs to be further explored and verified in subsequent work.

The PI3K/AKT signaling pathway can activate the mTOR signaling pathway. In the present work, it was also observed that COP treatment repressed the phosphorylation of mTOR. mTOR is capable of forming two distinct complexes, mTORC1 and mTORC2, and mTORC1 plays a key role in the regulation of autophagy [36, 37]. Under normal conditions, mTORC1 is activated, and it inhibits the formation of autophagy and initiation of autophagy by phosphorylating unc-51 like autophagy activating kinase 1 (ULK1) and its interacting proteins; in the state of cellular stress, the activity of mTORC1 is inhibited, and ULK1 is dissociated from mTORC1, and the ULK1 complex becomes active through autophosphorylation and promotes the formation of autophagosomes [36, 37]. In addition, mTORC1 can also indirectly inhibit autophagy by regulating transcription of genes required for lysosomal biogenesis [38]. In a variety of malignant tumors, the mTOR signaling pathway is hyperactivated, resulting in inhibition of autophagy, which promotes the growth and proliferation of tumor cells [39]. In this work, it was also found that COP could inhibit the phosphorylation of mTOR in a dose-dependent manner in T24 and BIU-87 cells, suggesting that COP may affect the progression of bladder cancer through modulating autophagy. This scientific hypothesis needs to be further verified by subsequent studies. Additionally, in our previous work, we have reported that COP represses the expression level of CCND1 in bladder cancer cells [16]. In the current study, our data further support that COP represses CCND1 activity via both repressing its expression and its binding with CCND1 protein. Considering CCDN1 is a crucial regulator in cell cycle progression and cell proliferation [40], our findings explain the reason that COP represses the growth of bladder cancer cells. Even though the detailed mechanisms by which COP represses the expression level of CCND1 in bladder cancer have not been fully explained, increasing evidence shows that CCND1 is positively regulated by PI3K/AKT pathway, mTOR pathway and GSK3β pathway [41–44], implying that the regulator network among COP, PI3K/AKT, and GSK3B contributes to the dysregulation of CCND1 in bladder cancer cells.

## Conclusion

AKT1, GSK3B, CASP3, TNF, CCND1 are the main targets of COP in the treatment of bladder cancer. COP has a therapeutic effect on bladder cancer by targeting the PI3K/AKT signaling pathway. This study provides a theoretical basis for the application of COP in bladder cancer treatment.

## Supplementary Information

Supplementary Material 1.

## Data Availability

All of the data and materials are available from the corresponding author upon reasonable request.
