# Supplementary material for: Coptisine regulates PI3K/AKT pathway to block bladder cancer progression: a study based on network pharmacology, in vitro and in vivo assays
Source: Hereditas. 2025 Nov 24;162:232. doi: 10.1186/s41065-025-00600-7 (PMC12642184; doi:10.1186/s41065-025-00600-7)

T24 cell:

p-ERK1/2


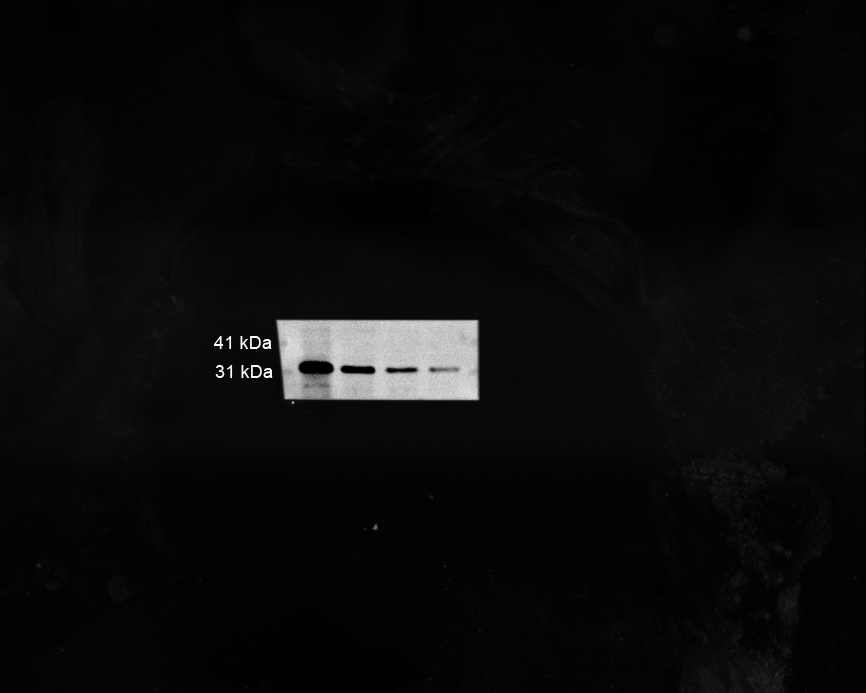


ERK1/2


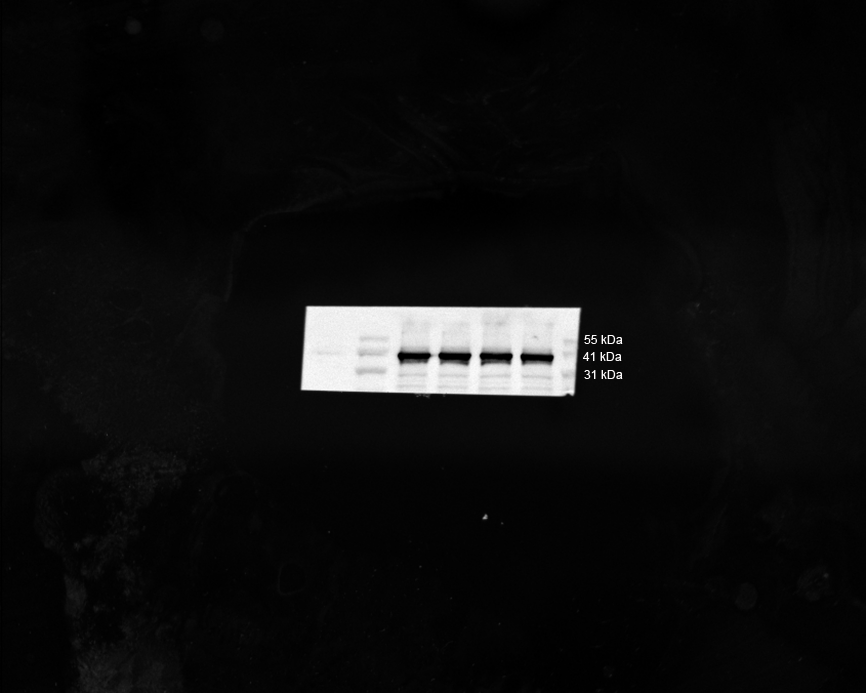


p-AKT


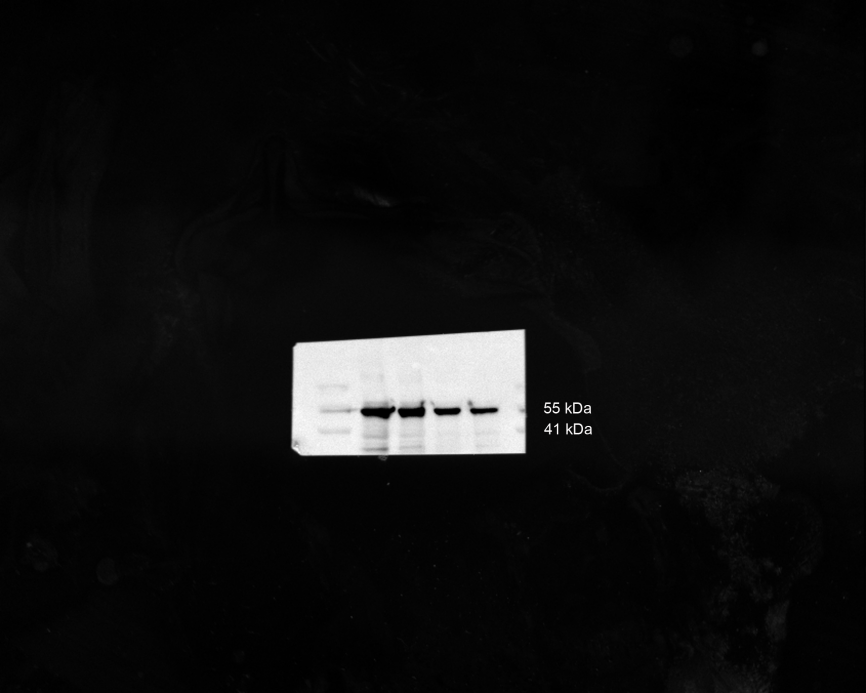


AKT


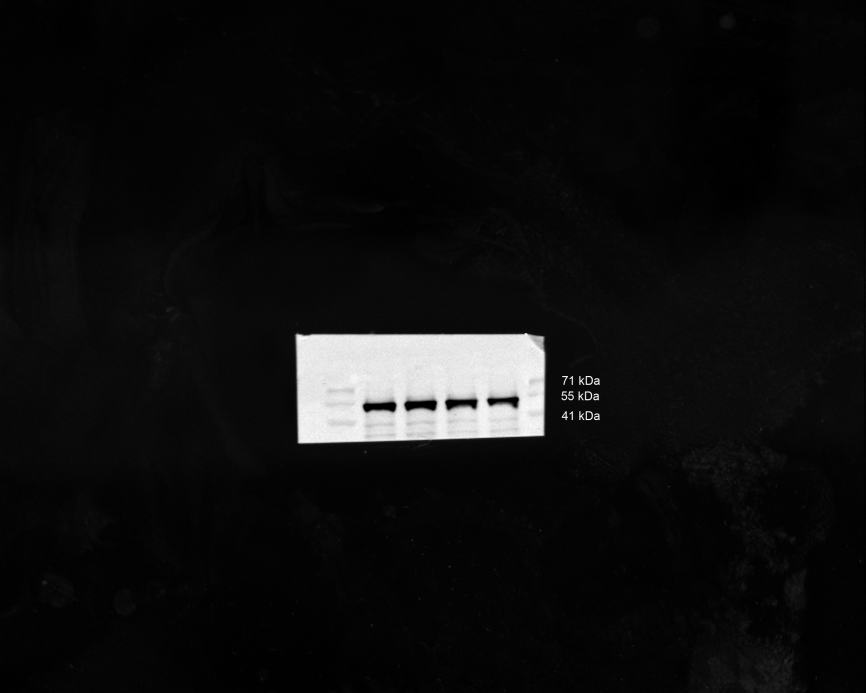


p-PI3K


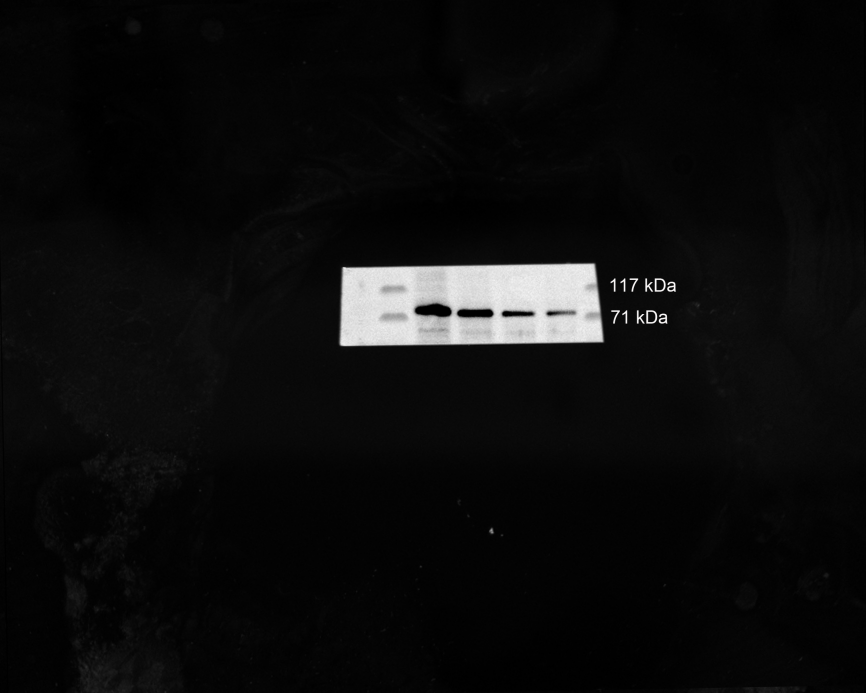


PI3K


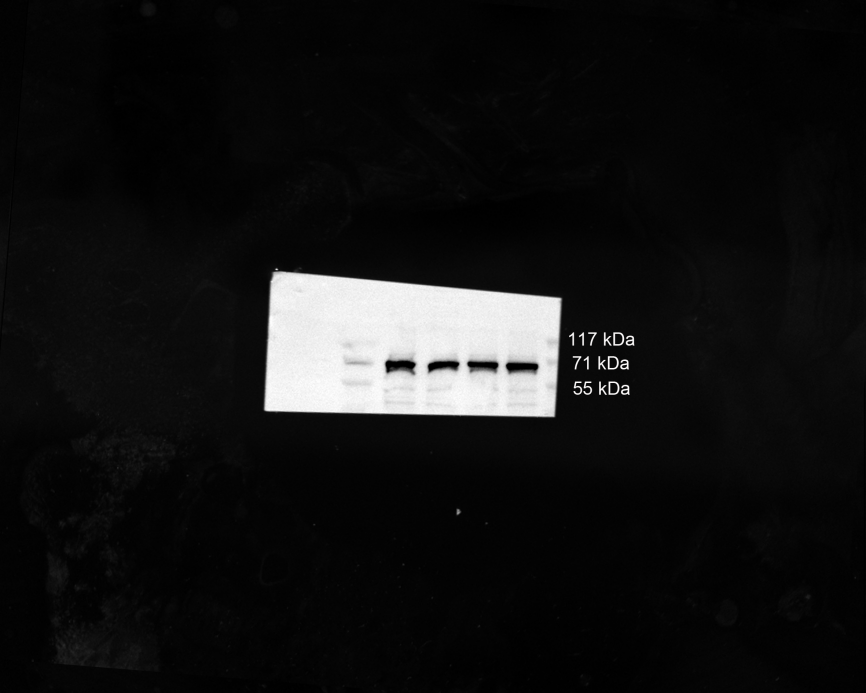


p-mTOR


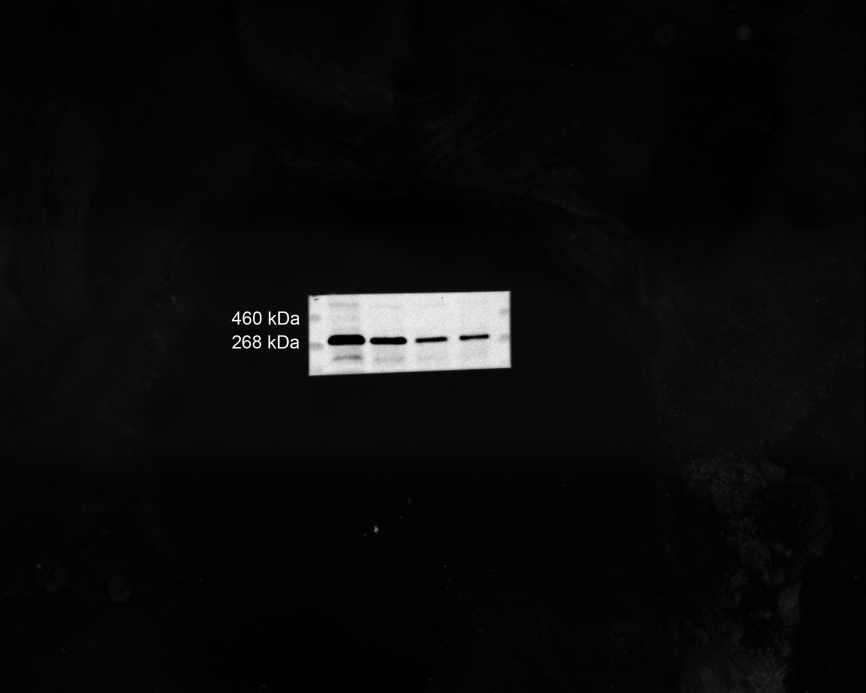


mTOR


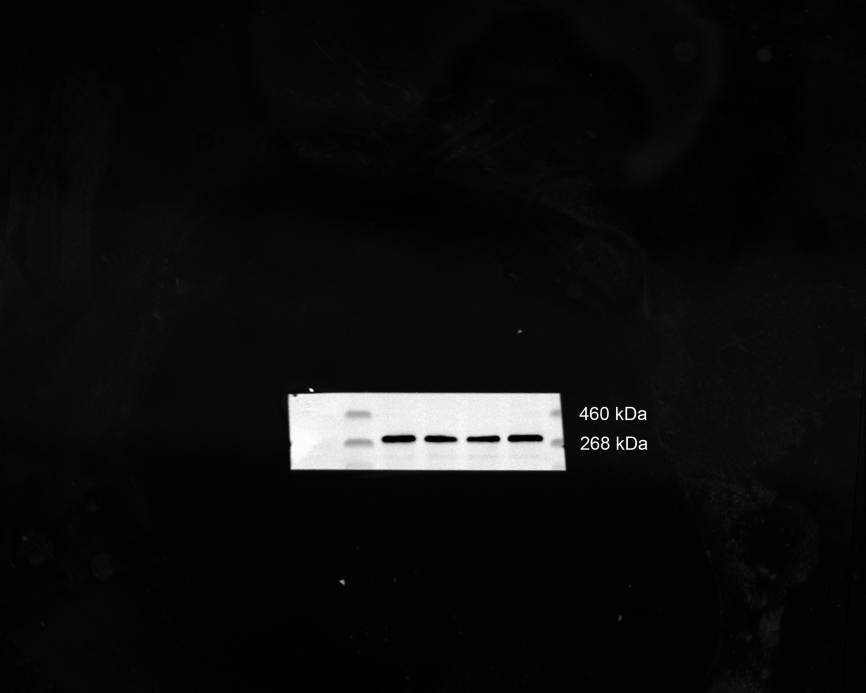


GAPDH


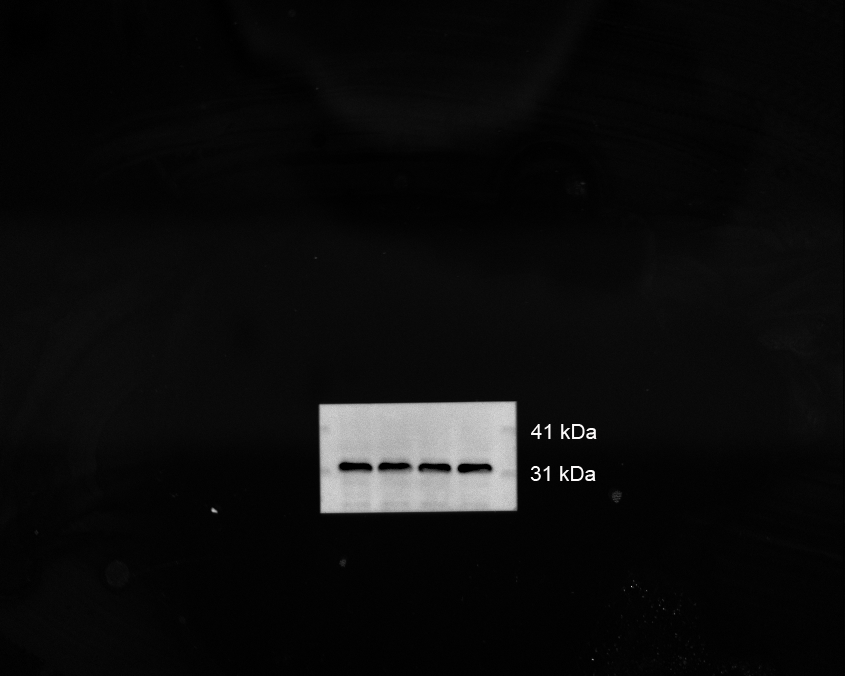


BIU-87 cell:

p-ERK1/2


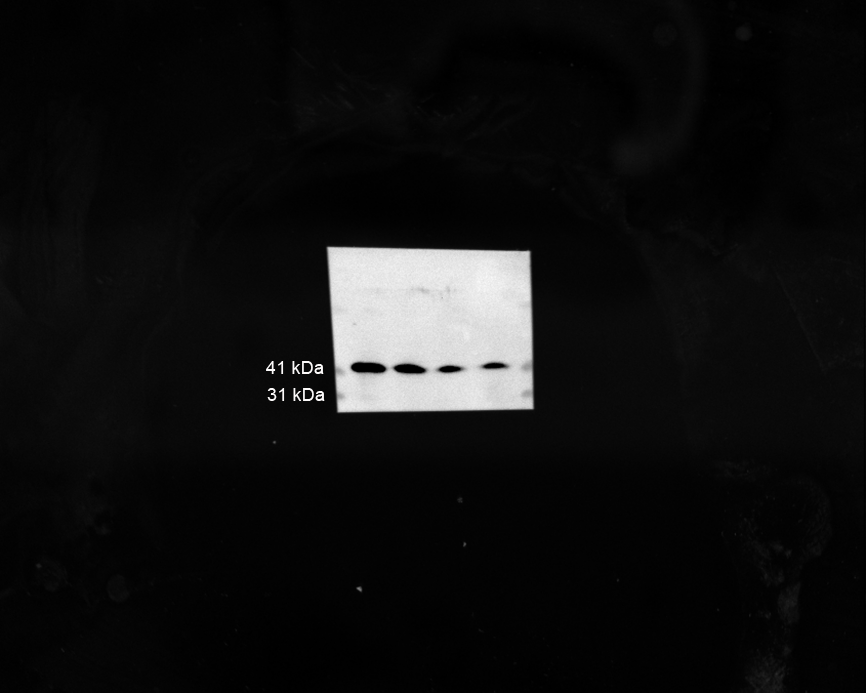


ERK1/2


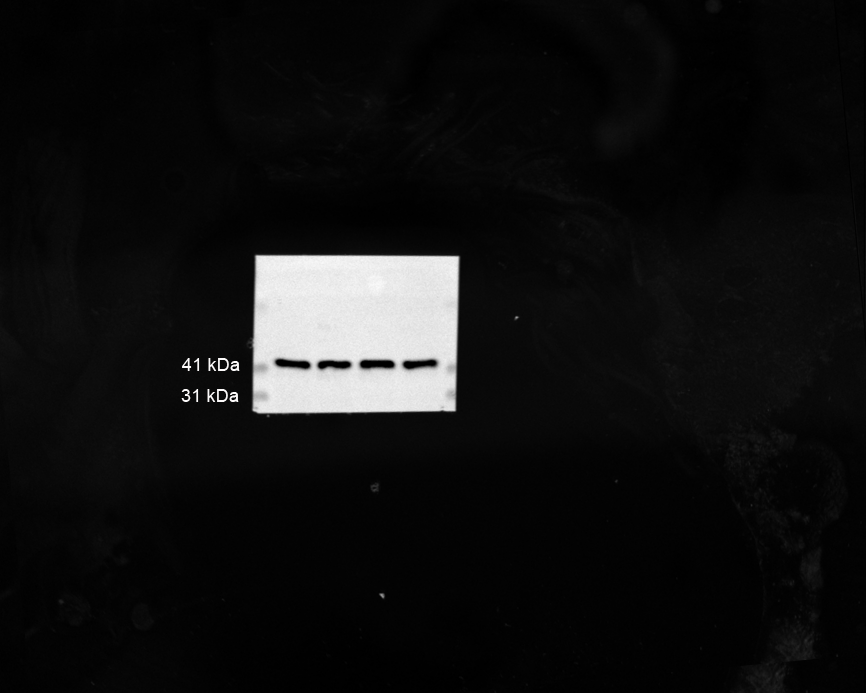


p-AKT


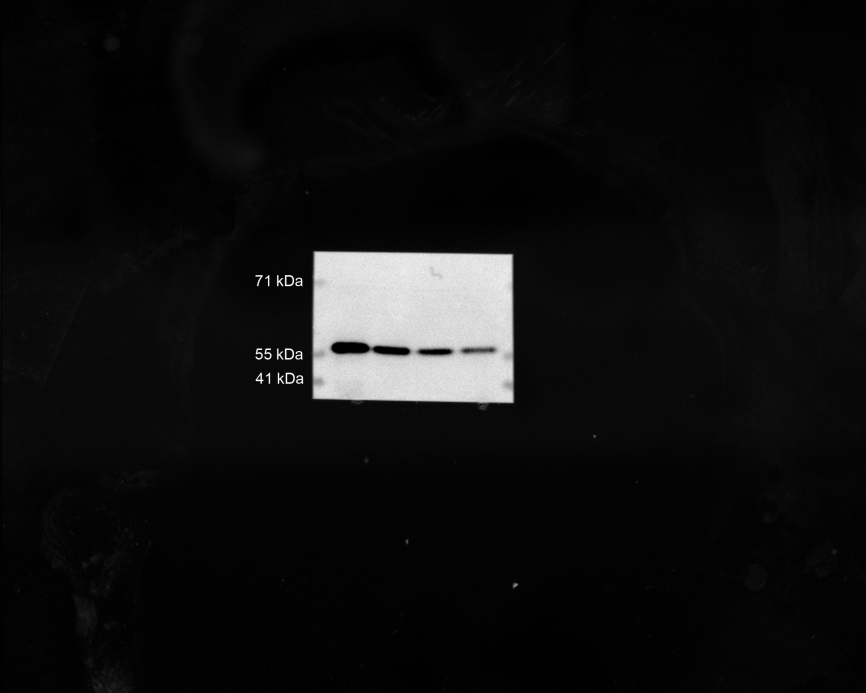


AKT


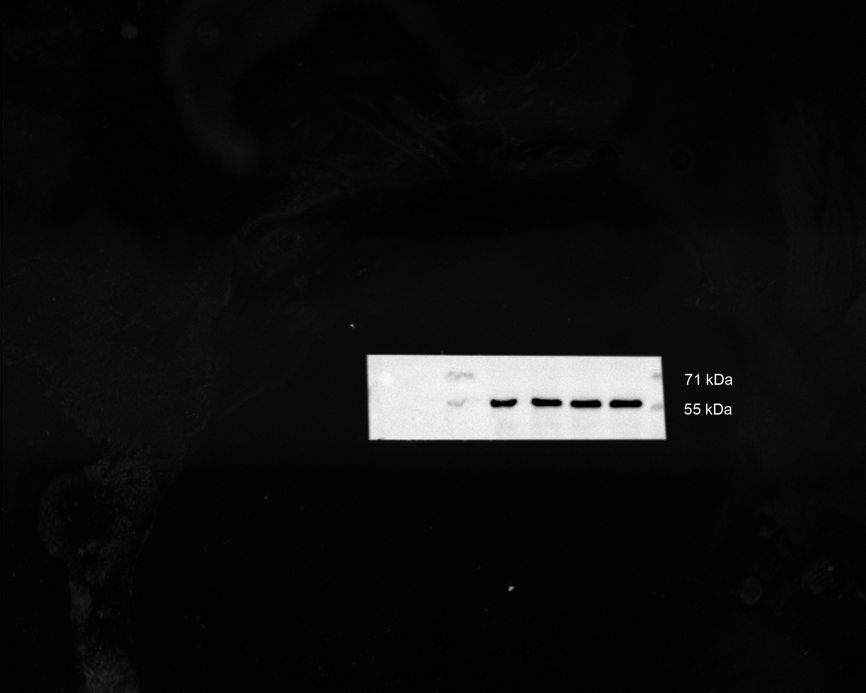


p-PI3K


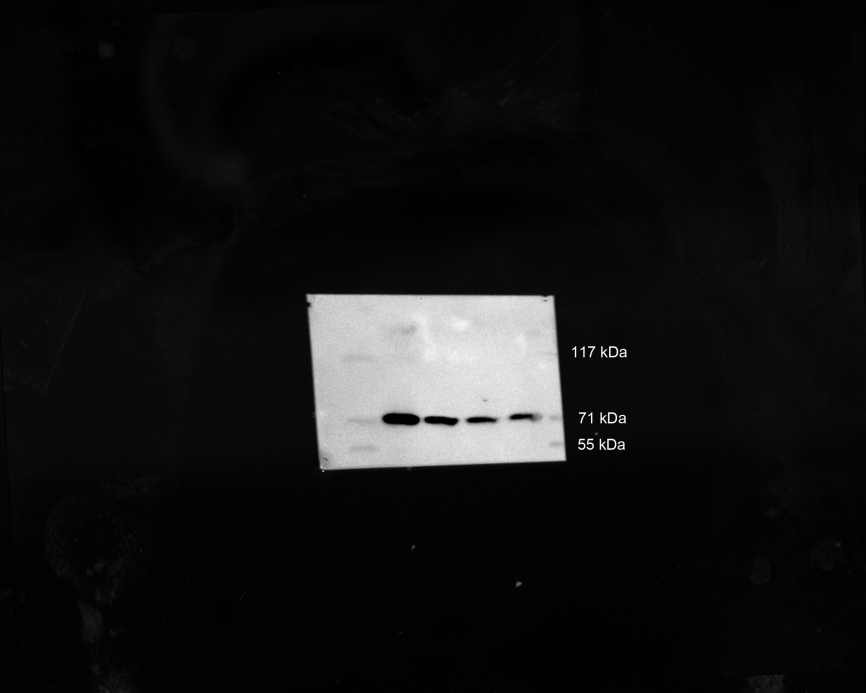


PI3K


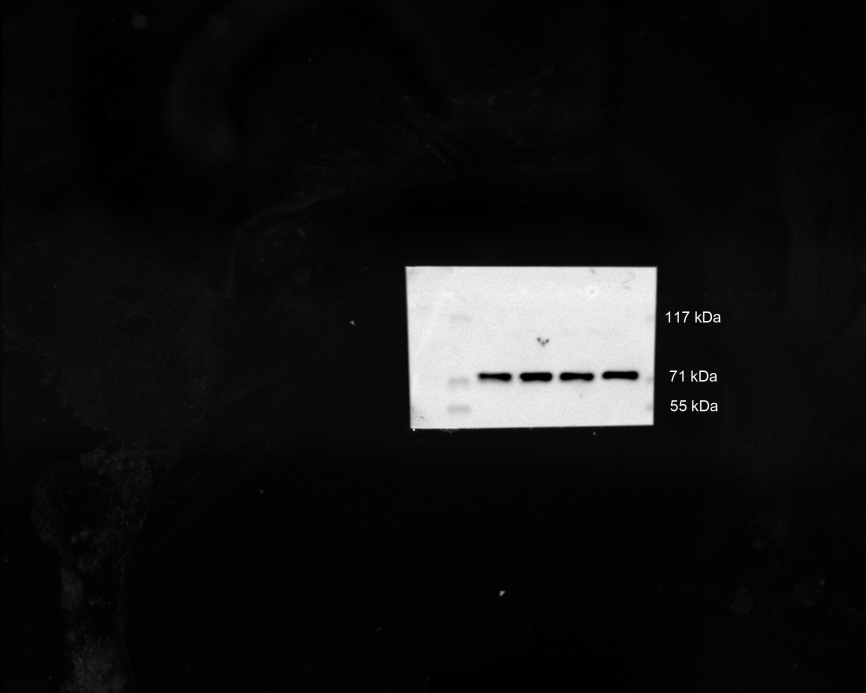


p-mTOR


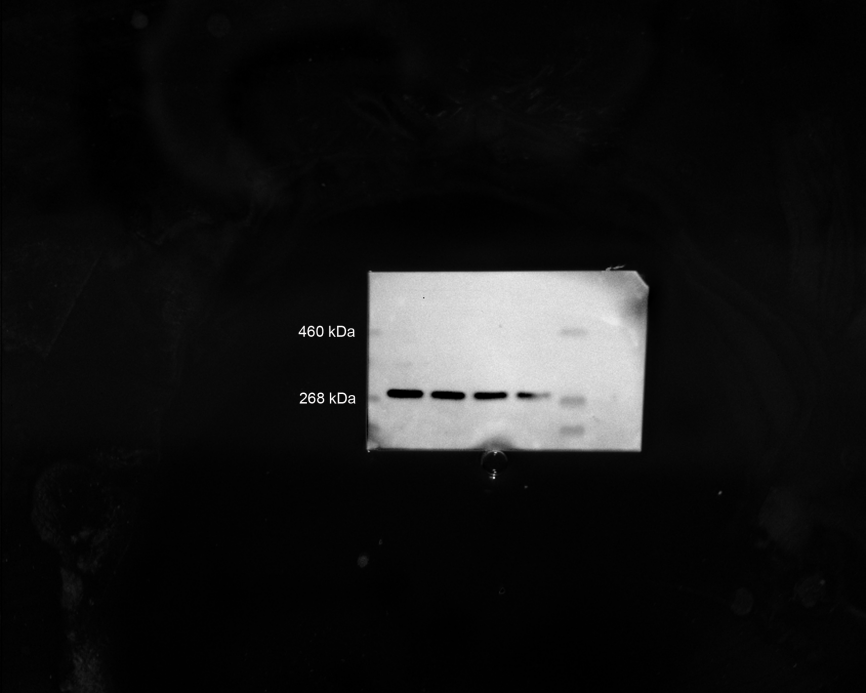


mTOR


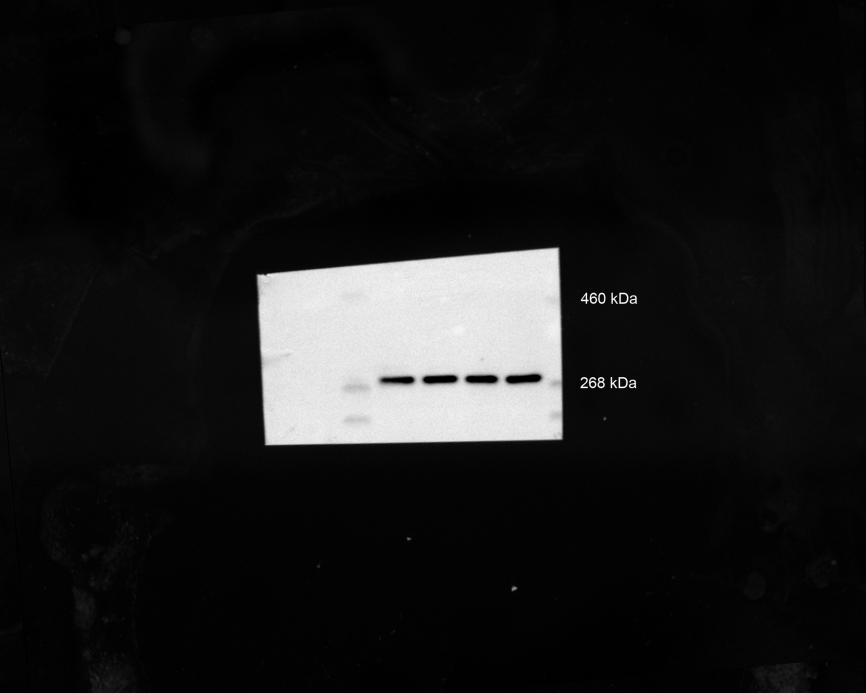


GAPDH


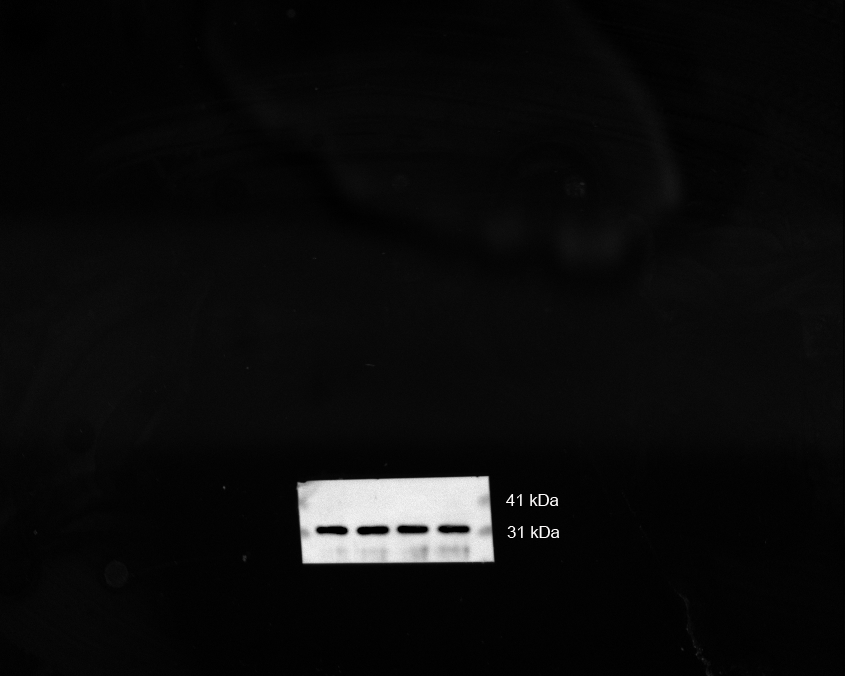

Supplement: Supplementary file 1 — Supplementary Material 1. [file 41065_2025_600_MOESM1_ESM.docx]
